# Digital health technologies in frontotemporal dementia: A scoping review

**DOI:** 10.1177/20552076261483445

**Published:** 2026-09-15

**Authors:** Liset de Boer, Olaiya A. Aro, Harro Seelaar, Lize C. Jiskoot, Jackie M. Poos

**Affiliations:** 1 Department of Neurology and Alzheimer Center, 6993 Erasmus MC University Medical Center Rotterdam, Rotterdam, NL

**Keywords:** frontotemporal dementia, digital health technologies, early diagnosis, remote monitoring, cognition

## Abstract

**Objective:**

Digital health technologies are increasingly used to assess cognition and behavior in neurodegenerative diseases. While such tools have shown promise in Alzheimer’s disease, including the preclinical stages, their use in frontotemporal dementia (FTD) remains underexplored. This scoping review provides an overview of the current literature on digital health technologies in FTD.

**Methods:**

We conducted a literature search according to the PRISMA-ScR guidelines and included 62 studies investigating digital health technologies in individuals with FTD.

**Results:**

The identified technologies included eye tracking paradigms, smartphone- and tablet-based cognitive assessments, and passive monitoring approaches such as wearable sensors and mobile applications. Most studies reported that these technologies could distinguish individuals with FTD from healthy controls or other dementia syndromes. However, many studies were experimental or feasibility-focused, and systematic evaluation of psychometric properties remains limited.

**Conclusion:**

In order to move the field forward, we argue that an in-depth investigation of the relationships between digital health technologies, classical pen-and-paper assessments of cognitive and behavioral functioning, and other biomarkers is necessary to establish clinical validity. Other important aspects to address in future research include the use of diverse cohorts and studying longitudinal effects. In our view, these next steps are crucial to advance digital health technologies toward implementation in clinical practice and clinical trials for FTD.

## Introduction

Frontotemporal dementia (FTD) is an early-onset neurodegenerative disorder affecting individuals at relatively young ages.^1^ FTD is characterized by progressive atrophy of the frontal and/or temporal lobes, leading to early impairments in amongst others social cognition, executive function, and language.^2,3^ Early in the disease course, people with FTD often exhibit subtle behavioral and cognitive alterations, such as loss of empathy, disinhibition, apathy, and language difficulties, that can precede clinical diagnosis by several years^4^ and put a significant burden on people with FTD and their families.^5^ These behavioral and cognitive changes are typically noticed by family members but are difficult to quantify with current diagnostic tools, often resulting in delayed diagnoses and misattribution to psychiatric conditions.^6^

Neuropsychological assessments are an important component of the clinical diagnostic process in FTD. However, multiple studies have demonstrated that traditional paper-and-pencil tests lack sensitivity to detect cognitive decline in the early stages of the disease,^3,6–9^ and often fail to differentiate FTD from other dementia types, such as Alzheimer’s disease (AD), or primary psychiatric disorders.^10^ This may be attributed to several factors. Firstly, these assessments are typically conducted in clinical settings using fixed stimuli and standardized tasks, which can be time-consuming and do not capture the complexity and variability of real-life situations and activities, thereby limiting their ecological validity.^11^ Secondly, they typically rely on outcome measures with limited variability, leading to ceiling effects—especially in early disease stages—that hinder detection of subtle deficits.^6,12^ Thirdly, traditional neuropsychological assessments provide only a single snapshot of an individual’s functioning at a specific point in time, thereby neglecting the potential daily fluctuations which is characteristic of early FTD.^13,14^ This is particularly problematic given the insidious onset and heterogeneity of symptoms. Lastly, because individuals with FTD often have limited insight into their own changes,^15^ early clinical assessment heavily depends on subjective reports from family members and clinicians’ impressions gathered during brief visits, which can be influenced by recall biases and subjective judgments. Collectively, these assessment-related limitations contribute to diagnostic delays and hinder early detection, effective monitoring in clinical trials, and tailored support after the diagnosis. Moreover, practical barriers such as long waiting lists for clinical appointments and limited access to specialized FTD centers can further delay assessments and interventions. Taken together, this underscores the urgent need for more sensitive, continuous, and ecologically valid assessment tools.

Digital health technologies have potential to address these limitations. Emerging digital tools, such as smartphone- or iPad-based assessments, wearable sensors, eye tracking devices, and other digital platforms, offer the possibility of continuous, objective, and real-world monitoring of cognitive and behavioral functioning.^16,17^ They can be administered in-person or remotely, that both come with several advantages compared to traditional neuropsychological tests. In-person digital health technologies could allow for more precise stimulus control and adaptation to an individual’s performance level, increasing efficiency and reducing assessment time and costs. In addition, they may improve accuracy and validity, as they can collect objective and quantifiable data and are potentially more accurate for some measures.^18^ Remote digital health technologies can collect longitudinal and passive data that is implemented in an individual’s natural environment, which can improve ecological validity and provide the opportunity to capture natural variability and fluctuations in behavior and cognitive functioning that are often missed during clinic visits.^13^ This could minimize recall biases and subjective judgments, as well as limit ceiling effects. Moreover, they enable widespread screening and monitoring, which is particularly important in remote areas where specialized centers are not readily accessible.^19^ This advantage substantially reduces waiting lists and travel burden for people with FTD and caregivers. Altogether, the rich and objective data generated by digital health technologies can improve early identification of neurodegenerative changes, facilitate personalized intervention strategies, and ultimately improve patient outcomes.

Despite these advantages, digital health technologies may also introduce challenges that should be considered. Remote assessments could reduce experimental control compared to supervised in-clinic testing, making it difficult to verify whether tasks are completed independently or under standardized conditions. Technical factors such as internet connectivity, device variability, software compatibility, and digital literacy may also influence performance, particularly for tasks relying on reaction times or precise timing measurements.^14,19^ In addition, passive or real-world monitoring approaches can generate large and complex datasets that require careful interpretation, and concerns regarding privacy and data security remain important considerations.^20^

Multiple digital technologies have already been developed. They have already shown their sensitivity to subtle cognitive changes in preclinical AD.^19^ However, a clear overview of digital health technologies developed for FTD is still missing. This scoping review seeks to comprehensively map the current landscape of digital health technologies used for assessing and monitoring behavior and cognition in FTD. It explores the range of in-person and remote technologies, summarize their findings, evaluate their feasibility and validity, describe strengths and shortcomings, and identify possibilities for future research to enhance early detection and monitoring of clinical presentations in the FTD spectrum.

## Methods

### Protocol and registration

The review protocol was preregistered on the Open Science Framework (OSF, doi:10.17605/OSF.IO/X4FRJ). The review methodology was guided by the PRISMA-ScR (Preferred Reporting Items for Systematic Reviews and Meta-Analyses extension for Scoping Reviews).

### Information sources and search strategy

To ensure a comprehensive and systematic literature search, we collaborated with the medical library from the Erasmus MC University Medical Center (Rotterdam, the Netherlands) to develop and execute the search strategy. An extensive literature search was conducted in MEDLINE, EMBASE, Web of Science, and Cochrane. The full search strategy is included in Supplementary File 1. The initial search was conducted on August 29, 2024, and updated on May 8, 2025, and January 29, 2026. We included a manual search by means of literature snowballing and consulting reference lists in previous reviews. We additionally conducted a targeted search to identify studies using wearable technologies in FTD that were not captured in the initial search strategy. This supplementary search used citation tracking and targeted keyword searches related to actigraphy, wearable sensors, and behavioral monitoring in FTD. Studies identified through this process that met the inclusion criteria were added to the review.

**Figure 1.**
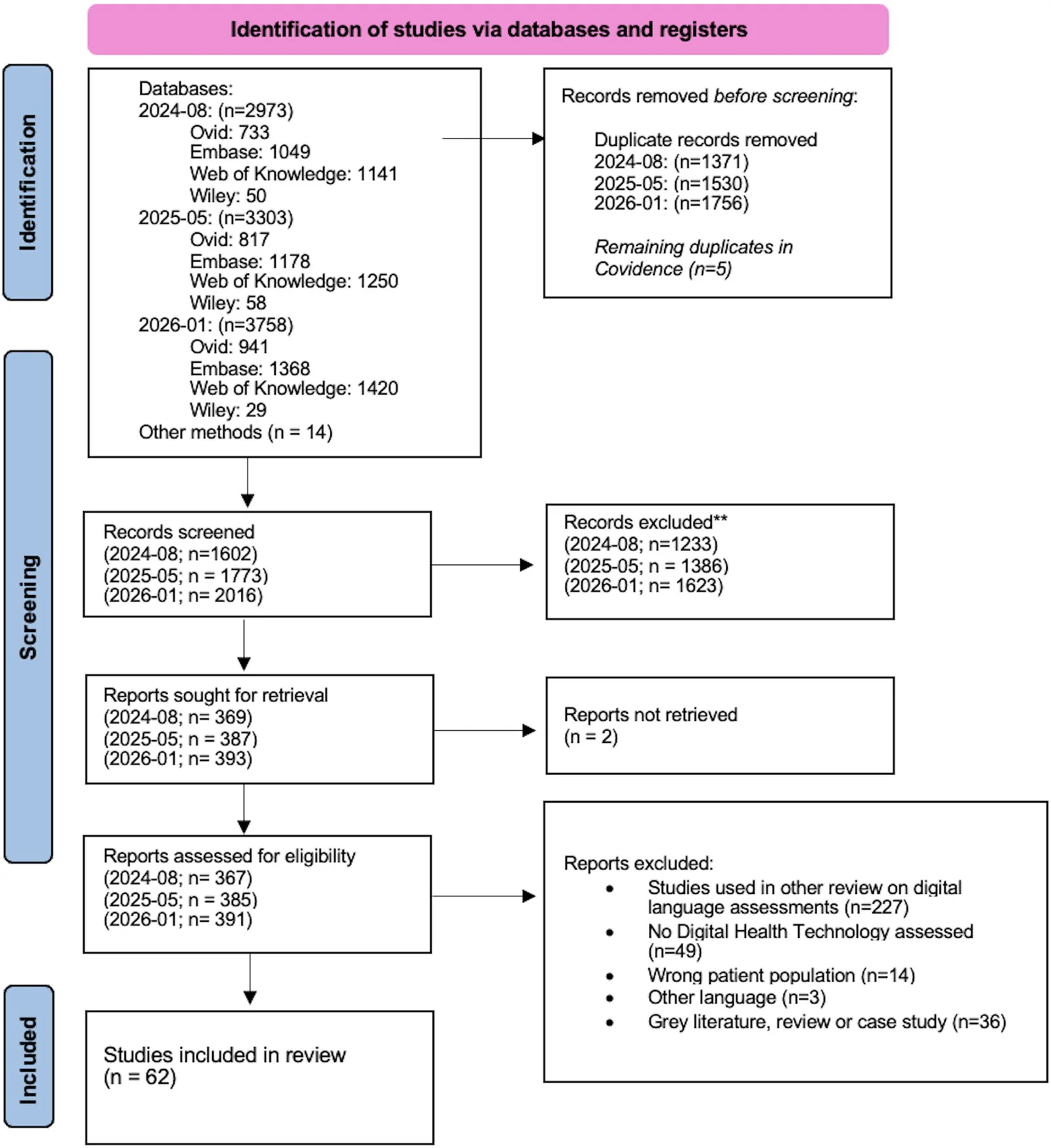
PRISMA flow diagram.

All records retrieved from the searches were imported into Covidence (www.covidence.org). Three reviewers (LdB, OA, JMP) independently screened all titles and abstracts of the retrieved records. Conflicts were discussed and resolved by all three reviewers. Two reviewers (LdB, OA) then independently assessed full-text articles. The study selection process and number of studies at each stage are displayed using the PRISMA flow diagram (Figure 1). Although automated speech and language analyses were included in the search strategy, studies focusing primarily on speech-based digital biomarkers were excluded from this review. The volume and distinct methodological considerations of this literature warranted a separate review. Accordingly, a dedicated review has been preregistered by OA on the Open Science Framework (OSF; doi:10.17605/OSF.IO/D473T).

### Eligibility criteria

We included empirical studies that used a digital health technology to directly assess cognitive or psychiatric/behavioral functioning in the context of diagnosis or monitoring in FTD-spectrum disorders (behavioral variant FTD, bvFTD; primary progressive aphasia, PPA; amyotrophic lateral sclerosis, ALS; progressive supranuclear palsy, PSP; corticobasal syndrome/degeneration, CBS/CBD) and presymptomatic carriers of pathogenic genetic mutations associated with FTD (e.g., chromosome 9 open reading frame 72 (C9orf72), progranulin (GRN), and microtubule-associated protein tau (MAPT)). Eligible technologies included, eye tracking paradigms, wearable devices, smartphone-based applications, and passive digital approaches, provided that the digital measures were specifically linked to the assessment of cognitive domains or psychiatric/behavioral symptoms (Table 1; Supplementary file 1). Passive digital measures (e.g., smartphone activity patterns) were also included when the study aimed to investigate whether real-world behaviors captured through these technologies relate to cognitive performance or specific behavioral domains. Motor, physiological, or activity-based measures were only included when they were acquired within a cognitive or behavioral assessment paradigm or were interpreted as direct indicators of cognitive or neuropsychiatric functioning, rather than solely as markers of motor- or physiological impairment. Included studies were required to report group differences, correlations with traditional cognitive or behavioral measures, and/or psychometric and clinical properties of the digital technology, including diagnostic accuracy, reliability, validity, feasibility, acceptability, or standardization (e.g., normative studies). An overview of eligibility criteria is reported in Table 1.

**Table 1.** Inclusion and exclusion criteria.

Criteria	Inclusion	Exclusion
Language	English	Any other language
Type of article	Primary research	Abstracts, commentaries, editorials, letters to the editor, case reports, case series, animal studies, pilot studies, duplicate studies
Population	Primary condition is one of: - Behavioral variant FTD (bvFTD; criteria Rascovsky et al.^21^ or Neary et al.^22^) - Primary Progressive Aphasia (svPPA, nfvPPA, lvPPA, criteria Neary et al.^22^ or Gorno-Tempini et al.^23^) - Amyotrophic Lateral Sclerosis (ALS; criteria El Escorial et al.^24^) - Progressive Supranuclear Palsy (PSP; criteria Litvan et al.^25^ or Höglinger et al.^26^) - Corticobasal Degeneration/Corticobasal Syndrome (CBD/CBS; criteria Armstrong et al.^27^ or Boeve et al.^28^) - Presymptomatic carriers of pathogenic genetic mutations associated with FTD (e.g., *C9orf72*, *GRN*, and *MAPT*)	Any other group.
Setting of administration	In-person or remote assessment. Digital health technologies should be assessed in the FTD spectrum. Digital health technologies that are designed for neurodegenerative diseases in general and that are studied in at least one condition in the FTD spectrum are also included.	​
Reporting measures	Any digital health technology measuring one or multiple aspects of behavioral or cognitive functioning: Cognitive domains: - Attention - Processing speed - Executive functioning - Language - Memory - Social cognition - Visuospatial functions Behavioral/neuropsychiatric domains^29^: - Hallucinations/delusions - Agitation - Depression - Fear - Euphoria - Apathy - Disinhibition - Irritability - Motor behavior - Sleep - Appetite	Measures not associated with either cognition or behavior, or substitutions of paper and pencil tasks on a computer screen were not included.
*Outcomes*	Provide cognitive or behavioral data, or outcomes of motor behavior and neuropsychiatric symptoms	Primary objective not specific to cognition, behavior, motor functioning, or neuropsychiatric symptoms.

We excluded studies in which the clinical sample was not composed of individuals with a condition within the FTD spectrum. Studies were not considered if digital measures solely captured motor, physiological, or activity-related features without a clear and direct assessment of cognitive or psychiatric/behavioral functioning. In addition, grey literature (e.g., abstracts, conference proceedings, editorials, clinical notes), reviews, and case studies were excluded. Studies focusing exclusively on imaging or machine learning, without a clear application to digital health technologies, were not included.

### Data items

The following data features were extracted from each included study:

1. Study characteristics: author(s), year of publication, journal of publication, and country of study.
2. Population characteristics: sample size, diagnostic group, and disease stage (if reported).
3. Type of assessment: type of digital technology used, the name of the assessment or platform, setting (remote vs. in-person or clinic based).
4. *C*ognitive or behavioral domains assessed: includes evaluations from all cognitive domains (executive function, memory, language, social cognition, attention, processing speed, visuospatial function) and behavioral domains (based on the behavioral domains of the Neuropsychiatric Inventory (NPI)^29^).
5. Key findings: includes the main results of the digital health technology.
6. Psychometric properties (if reported): includes reliability (e.g., test–retest, internal consistency), validity (e.g., construct, convergent, incremental, discriminant, criterion), diagnostic accuracy, and normative properties.
7. Implementation properties: includes feasibility and acceptability (e.g., adherence, usability)
8. Challenges and limitations: reported issues such as technical difficulties, sample sizes, excluded participants, missing data, practice effects, device-related variability, methodological limitations, sample bias, and recommendations for future research.

### Data extraction and synthesis

Data extraction was performed on the included articles by two reviewers (LdB, OA) by using a developed data extraction sheet. This sheet included details related to the data items described above. Studies were categorized according to the type of digital assessment, based on whether the assessment required active task engagement and whether it was conducted in-person or remotely. Active assessments refer to structured tasks requiring participants to actively perform a test (e.g., digital cognitive tasks or eye tracking paradigms), whereas passive assessments involve unobtrusive data collection without explicit task demands (e.g. step count, smartphone use). Assessments were further classified as in-person when conducted in a supervised laboratory or clinical setting using dedicated equipment, or remote when data were collected outside these settings using personal or portable devices.

### Patient and public involvement

Individuals were not involved in the design or completion of this study.

### Definitions of eye tracking measures

Because eye-tracking studies included in this review used a wide range of oculomotor paradigms and outcome measures, we briefly describe the most commonly reported eye-tracking metrics and their relevance to cognitive and behavioral functioning (Table 2). Common eye tracking outcomes include several measures of eye movement and ocular responses. *Saccades* are rapid eye movements that shift gaze between locations and can be stimulus-driven (prosaccades or visually guided saccades^30,31^), inhibitory (anti-saccades^31^), self-paced (self-initiated eye movements made between two fixed targets^32^), memory-guided (directed to a previously seen location), predictive (saccades that anticipate the future position of a target^33^), or delayed (participants must withhold an eye movement until a cue is given^34^). These are often quantified using metrics such as latency, amplitude, velocity, and accuracy.^31^ *Fixations* refer to periods during which gaze remains relatively stable and visual information is processed, typically measured by fixation duration or number of fixations.^17^ *Dwell time* represents the total time spent looking at a specific stimulus or area of interest and may include multiple fixations within that region.^35^ *Smooth pursuit* describes continuous eye movements used to track moving objects and is commonly assessed through tracking accuracy or pursuit gain.^36^ *Visual search* measures evaluate how efficiently individuals locate targets among distractors and include metrics such as search time, number of fixations, scan path patterns, and time to first fixation on the target.^37^ *Square-wave jerks* are brief, involuntary saccadic intrusions away from and back to a fixation point.^38^ Finally, *pupil dilation* reflects changes in pupil size that can index cognitive effort, arousal, or autonomic responses during task performance.^39^

**Table 2.** Eye tracking measures, their definitions, and the cognitive/behavioral domain assessed.

Eye tracking measure	Definition	Cognitive/behavioral domain assessed
Prosaccades/visually guided saccades	Reflexive eye movements toward a visual stimulus	Attention, visuospatial orienting, processing speed
Anti-saccades	Eye movements requiring inhibition of a reflexive gaze toward a stimulus	Executive function, inhibitory control
Self-paced saccades	Voluntary eye movements made between fixed targets	Initiation, motor planning
Memory-guided saccades	Eye movements directed toward a previously viewed location	Working memory, spatial memory
Predictive saccades	Eye movements anticipating target movement	Prediction/anticipation, executive control, attentional processing
Delayed saccades	Eye movements initiated only after a cue	Response inhibition, executive control, sustained attention
Saccade accuracy/amplitude/velocity	Spatial and dynamic characteristics of eye movements	Motor control, visuospatial processing, executive functioning
Fixations	Stable gaze periods during visual information processing	Attention, information processing, social cognition
Dwell time	Total viewing time on a stimulus or area of interest	Attention, emotional processing, social cognition
Smooth pursuit	Continuous tracking of moving targets	Sustained attention, attentional tracking, executive control
Visual search	Efficiency of locating targets among distractors	Attention, executive functioning, visual exploration, processing speed
Square-wave jerks	Involuntary saccadic intrusions during fixation	Oculomotor control, attentional stability
Pupil dilation	Changes in pupil size during task performance	Cognitive effort, arousal, emotional processing

## Results

**Figure 2.**
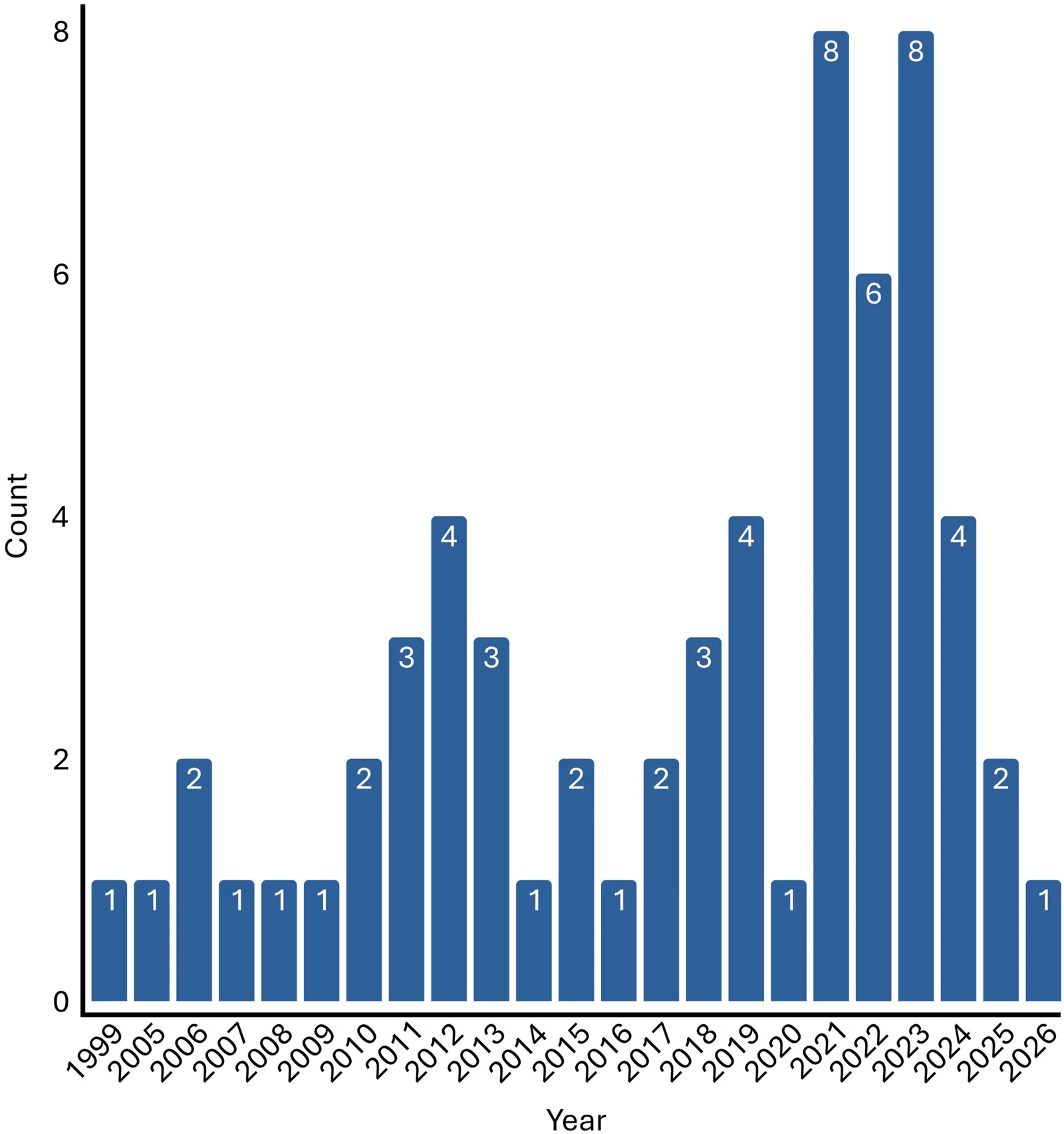
Histogram of the number of studies published per year.

**Figure 3.**
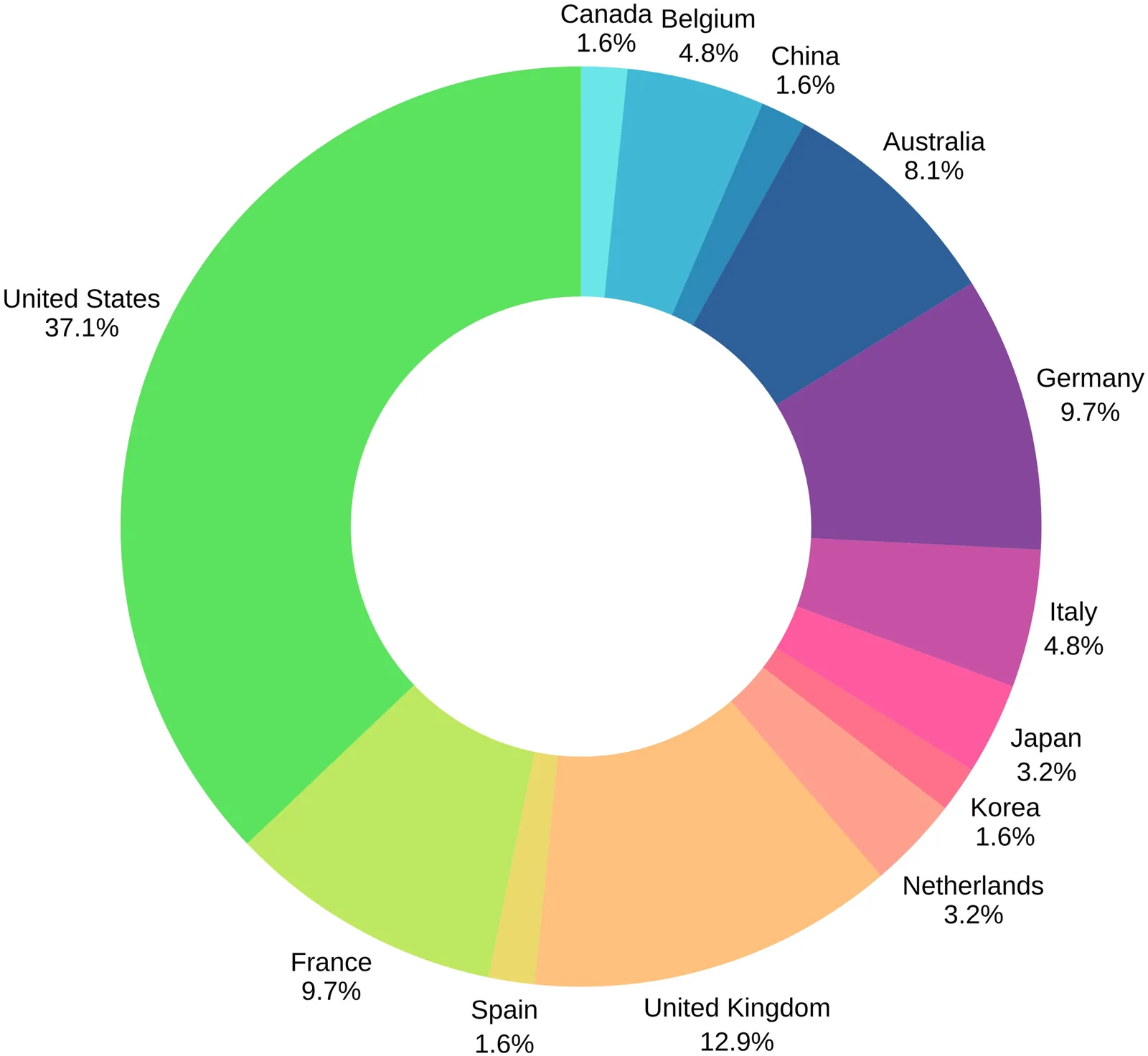
Overview of the number of studies published per country.

The systematic searches of the databases identified 3772 articles. After duplicates were removed and title and abstract screening (n=2016) were complete, 391 articles remained for full-text review. On full-text review, 62 studies met all criteria and were included (Figure 1). Figure 2 shows the number of included studies per year of publication. Figure 3 shows the number of included studies published per country.

Overall, most studies were published in the past decade, with a noticeable increase during the COVID-19 pandemic, reflecting the growing interest in digital biomarkers for neurodegenerative diseases. Most studies were conducted in America and Europe and most frequently involved individuals with bvFTD. The largest proportion of studies focused on in-person active assessments, particularly eye tracking tasks (Table 3). A smaller number of studies explored remote active assessments, such as tablet- or smartphone-based cognitive tests, while remote passive approaches, including wearable devices and smartphone-derived behavioral data, were less commonly studied but represent a developing area. The majority of studies focused on identifying group differences, while relatively few assessed psychometric properties, including measures of validity, reliability, or diagnostic performance (Supplementary Table 1).

**Table 3.** Characteristics of included studies.

Population	N (%)
bvFTD	34 (54.8%)
svPPA	23 (37.1%)
nfvPPA	12 (19.4%)
ALS	11 (17.7%)
PSP	19 (30.6%)
CBS/CBD	8 (12.9%)
Presymptomatic/prodromal	5 (8.1%)
**Setting of administration**	
In-person active assessment	52 (83.9%)
*Eye tracking*	*48 (77.4%)*
*Other*	*5 (8.1%)*
Remote active assessment	4 (6.5%)
Remote passive assessment	6 (9.7%)
**Study design**	
Cross-sectional	52 (83.9%)
Longitudinal	7 (11.3%)
Feasibility study	4 (6.5%)
Validation/normative study	9 (14.52%)

*Note.* Studies including multiple populations and study designs were counted in each relevant category; therefore, percentages may exceed 100.

### In-person active assessment

#### Eye tracking

Among the upcoming technologies, eye tracking has gained significant interest as a tool for detecting cognitive dysfunction in FTD. Across FTD phenotypes, eye tracking studies revealed distinct patterns of oculomotor and visual processing abnormalities that are indicative of a range of cognitive domains including language, memory, attention and executive function and social cognition. In bvFTD, the most consistent finding is impaired anti-saccade performance, with higher error rates than healthy controls, although individuals with bvFTD often correct these errors more frequently than patients with other neurodegenerative disorders such as AD or PSP.^17,34,40–46^ Findings for prosaccades are less consistent, as some studies report increased latencies,^17,34,44^ while others found no significant differences compared to healthy controls.^40,42^ Impairments have also been observed in predictive and memory-guided saccades^40,44,47^ and smooth pursuit.^36,42^ In visual search tasks, bvFTD is characterized by reduced efficiency, reflected by increased fixation counts, longer fixation durations, and slower response times.^37,48^ Social cognition paradigms further show altered gaze allocation during emotion recognition tasks, including atypical fixation patterns on facial features and reduced attention to emotionally salient stimuli.^35,49–53^ One study reported an eye tracking task assessing executive functioning based on a traditional neuropsychological test and found that individuals with bvFTD, compared to people with svPPA and controls, had fewer correct and more incorrect anticipations.^54^ In presymptomatic carriers of FTD-related pathogenic variants, limited evidence suggests subtle early abnormalities, including increased predictive saccade latencies and elevated anti-saccade error rates, although these findings are based on small samples.^47,55^ Finally, two studies examining pupillary responses during cognitive tasks found preserved responsiveness to cognitive effort, but reduced dilation magnitude compared to healthy controls.^39,56^

In svPPA, performance on basic saccadic and visual search tasks is generally preserved,^40–42^ but language-based paradigms reveals clear abnormalities: individuals with svPPA show reduced fixation on target images and increased attention to semantically related distractors during naming and word–picture matching tasks, reflecting degradation of semantic knowledge and associated temporal pole atrophy.^57–62^ In nfvPPA, impairments have been observed in comprehension of time reference 63 and sentence-level processing tasks, where reduced fixations on target images during thematic integration tasks suggest difficulties integrating complex syntactic information.^64^

Motor-disorders on the FTD spectrum such as ALS, PSP, and CBS are associated with characteristic abnormalities in saccades, smooth pursuit and executive oculomotor tasks. For example, several studies showed that individuals with ALS display increased early saccades,^65^ increased anti-saccade errors, abnormal visual search performance,^32,66,67^ and a higher number of fixations and saccades during an eye tracking version of the Trail Making Test (TMT).^66^ Studies of CBS demonstrated impairments in performance on an anti-saccade tasks.^41,46^ Studies of PSP similarly reported marked impairments in fixations and square wave jerks,^38,68,69^ saccades,^41,70–74^ and disrupted smooth pursuit.^75–77^ In one study, saccade velocity during saccadic tasks (prosaccade, anti-saccade, smooth pursuit) was significantly superior to neuropsychological tests (Trail Making Test) in differentiating PSP from other disorders.^41^

The included eye tracking studies reported several psychometric properties supporting the validity and potential clinical utility of this digital technology. Construct validity was mostly examined through correlations with standard neuropsychological measures. Anti-saccade performance and other executive eye tracking measures were consistently associated with executive functioning.^17,34,42,55,67,78,79^ Several studies also demonstrated neuroanatomical validity through correlations between oculomotor abnormalities and structural or functional brain changes, including frontal eye fields, midbrain volume, white matter tracts, and functional connectivity.^17,35,37,40,42,43,45,51,54,76^ Diagnostic accuracy was reported in several studies, particularly for differentiating PSP from other neurodegenerative disorders, with area under the curve values ranging from 0.70 to 1.00.^36,38,41,45,47,70,72^ Some studies further reported incremental validity of eye-tracking measures over conventional neuropsychological testing^41^ and feasibility indicators such as high task-completion rates in ALS populations.^66^

Several limitations were reported across the included studies. Small sample sizes and heterogeneous clinical cohorts were the most common limitations.^17,35,39,47,49,55,61,63,64,72,74^ Many studies also noted the absence of intensive neuropsychological testing or longitudinal follow-up, limiting conclusions regarding sensitivity to disease progression and cognitive specificity.^44,55,65,75,76^ Several authors also emphasized the potential value of applying these methods in early or presymptomatic stages of FTD.^40,49^ Technical challenges included recording noise, excessive data loss, head movement artifacts, limited precision of some eye-tracking techniques, and exclusion of participants with poor-quality recordings or ophthalmological conditions.^37,38,47,48,68,73^ Several authors also highlighted other potential patient-related confounding effects of medication use, fatigue, executive dysfunction, or task comprehension difficulties.^35,40,44,66,74^ Additional concerns included cross-sectional study designs,^17,79^ limited ecological validity of social cognition paradigms,^51,52^ and ceiling effects.^37^ Most studies, except for one eye tracking study,^80^ relied on structured, laboratory-based setups, with only limited use of portable systems, highlighting a lack of evaluation in real-world settings.

#### Computer/tablet-based cognitive assessment tools

Several digital cognitive assessment platforms included individuals with FTD during in-person experiments. The Cambridge Neuropsychological Test Automated Battery (CANTAB) is a neuropsychological battery used in studies examining FTD and demonstrates cognitive deficits in FTD that are not usually identified using traditional neuropsychological tests (e.g. patients performed normally on standard tasks such as spatial working memory but showed clear impairments on a digital decision-making task).^81^ More recently, Hammers et al. demonstrated that the CogState cognitive battery is able to discriminate dementia groups from patients with mild cognitive impairment (MCI) and healthy controls.^82,83^ The Tablet-Based Cognitive Assessment Tool (TabCAT)-EXAMINER is a tablet-based executive functioning battery and has normally distributed scores which were associated with disease severity and diagnosis.^84^ Baseline disease severity was associated with lower executive function scores and predicted faster longitudinal decline, indicating that the TabCAT-EXAMINER is sensitive to executive dysfunction and disease progression in FTD.^84^

The Cogstate study showed acceptable construct validity and short-term test-retest reliability with minimal practice effects.^82,83^ Test performance correlated with tests for visual form discrimination and visual reproduction. However, its ability to differentiate between dementia subtypes (including FTD) is limited.^82,83^ The (TabCAT)-EXAMINER study demonstrated construct validity and high test–retest reliability across a large cohort of older adults and individuals with neurodegenerative diseases.^84^ TabCAT-EXAMINER scores were correlating with tests for executive functioning.

Reported limitations across the studies included small sample sizes,^82,83^ heterogeneous disease stages,^82,84^ and limited cultural and demographic diversity.^84^ In addition, the Cogstate study reported exclusion of participants due to poor completion rates.^82^

#### Virtual reality

Initial steps toward the application of VR in cognitive and behavioral assessment for FTD have been made. Only one feasibility study has been published that refers to VR as a cognitive tool for assessing FTD.^85^ The authors of this study state that people with bvFTD showed their presence in the VR environment by moving their heads and responding to questions from the avatars in the VR environment.^85^ None of the people endorsed any physical complaints or discomfort with VR. This study reported a small sample size as limitation.^85^

### Remote active assessment

#### Eye tracking

One eye tracking study reports the use of a portable eye tracker in a home setting.^80^ They indicate that eye tracking may serve as a marker for predicting which words are more likely to be preserved or lost over time in individuals with PPA. Participants named pictures while their eye movements were recorded. More visual fixations and greater visual fixation dispersion during the task predicted which items were most likely to drop out at follow-up.^80^ However, reduced experimental control and precision were noted as key limitations.

#### Smartphone-based cognitive assessments

The ALLFTD Mobile Phone App is a smartphone-based neuropsychological assessment, designed to remotely examine cognition through short tests.^86,87^ Preliminary analyses suggested that the app’s cognitive tests were sensitive to early disease severity, whereas the MoCA did not detect these early differences. Another study evaluated the feasibility of smartphone-based digital phenotyping in individuals with ALS using the Beiwe platform, which collects active data (e.g., surveys and speech recordings) and passive smartphone sensor data.^88^ The smartphone-reported scores closely matched clinic-based measurements, demonstrating the potential of digital phenotyping for remote monitoring of disease progression.

The ALLFTD group reported good psychometric properties (internal consistency, test-retest reliability, construct validity, neuroanatomical validity, and diagnostic accuracy)^87^ and demonstrated that individuals across the FTD spectrum were generally able to complete the ALLFTD-App on their smartphones, with symptomatic participants completing 74.5% of tasks.^87^ Most participants found the instructions clear, although difficulties were mainly reported by prodromal or symptomatic individuals, including two with svPPA.^86^ The Beiwie platform shows good construct validity and feasibility, as participants were able to complete weekly remote assessments.^88^

The studies described above highlight several limitations and future directions for implementation of active remote assessments in clinical settings. First, they emphasize the need for longitudinal analyses and burst testing (e.g., a week of daily assessments every month)^87^ and cultural adaptations of the app.^86,87^ Second, the studies report possible complications in software (e.g. exclusion of participants due to errors or task crashes).^86^ Third, these studies have not yet explored differences across dementia syndromes (e.g. differences between bvFTD and other forms of dementia both within and beyond the FTD spectrum) or assessed their ability to accurately differentiate between them.

### Remote passive assessment

#### Wearable technologies

Wearable technologies have been used to quantify real-world behavior in FTD. Several studies used wrist-worn actigraphy measuring activity levels, sleep, circadian rhythms, apathy, and agitation. Early studies demonstrated that actigraphy could objectively capture agitation and motor activity abnormalities in dementia populations including FTD,^89^ while subsequent work showed disrupted rest–activity rhythms and sleep–wake patterns in FTD.^90,91^ They found that apathy was associated with lower levels of activity and higher levels of immobility.^90^ More recent studies have further examined activity patterns, sleep disturbances, and circadian alterations using wearable accelerometers and actigraphy^^92,93^^ However, a validation study comparing actigraphy with polysomnography in bvFTD found that actigraphy systematically overestimated total sleep time, sleep latency, and sleep efficiency while underestimating wake after sleep onset, highlighting important measurement biases that should be considered when using actigraphy to study sleep in this population.^92^

These studies report several psychometric and clinical properties supporting the potential utility of wearable technologies for monitoring behavioral and cognitive changes in FTD. Construct validity was demonstrated through associations between passive digital measures and established neuropsychiatric or cognitive assessments. For example, lower activity levels measured with actigraphy were associated with higher apathy and caregiver distress scores on the Neuropsychiatric Inventory,^90^ while increased daytime activity was associated with greater agitation and lower MMSE scores.^89^ One study also reported neuroanatomical validity, including associations between sleep disturbances and cortical thickness.^93^ The study of Tamburrino et al.^92^ evaluated the accuracy of actigraphy against polysomnography and found high sensitivity (93–96%) but lower specificity (57–62%) for sleep-related measures.

Limitations of these studies included small cohorts and the need for further validation in larger samples and over longer monitoring periods to be able to report conclusions about long-term adherence and clinical utility.^90–93^ Some studies lacked healthy control groups or comprehensive behavioral assessments, reducing interpretability and generalizability.^89,90^ Technical and implementation-related challenges included device non-wear,^91^ incomplete assessments,^93^ and equipment malfunction.^92^

#### Passive data via smartphone-based cognitive assessments

The ALLFTD app and the Beiwe platform capture passive data on smartphones that may reflect real-world behavioral functioning and clinical decline.^88,94^ For example, the Beiwe platform collects passive data that was related to mobility, movement patterns, phone use behavior, and location tracking.^88^ The ALLFTD group used battery percentage as a proxy for smartphone engagement and daily activity patterns.^94^ Individuals with an FTD phenotype showed less change in battery percentage compared to controls, which was associated with worse performance in all cognitive domains assessed and an increased number of neuropsychiatric symptoms.

The ALLFTD group demonstrated construct validity through associations between passive smartphone-use metrics and neuropsychological performance across several domains.^94^ They also report associations between smartphone-use behavior and gray matter volume.

Limitations included possible impact of hardware, software, and service connection on battery life.^94^ Additional concerns included limited demographic diversity and the need for longer-term monitoring.^94^

## Discussion

This scoping review highlights the growing interest in digital health technologies and provides a systematic overview of research involving such technologies within the FTD spectrum. Most studies focused on in-person active assessment, particularly eye tracking, which identified abnormalities in executive functioning, attention, language, and social cognition across the FTD spectrum. Fewer studies examined remote active and passive approaches, including tablet- and smartphone-based cognitive assessments and wearable monitoring technologies. Across modalities, studies reported promising psychometric properties, including construct validity, neuroanatomical validity, diagnostic accuracy, feasibility, and test–retest reliability. However, psychometric evaluation remained limited overall, and many studies were constrained by small sample sizes, cross-sectional designs, heterogeneous cohorts, and limited clinical validation. Taken together, these findings suggest that digital technologies show promise as scalable and sensitive tools for assessing cognitive and behavioral dysfunction in the FTD spectrum, but several gaps and limitations have been identified and are discussed in detail below.

### In-person active assessments

Studies suggest that eye movement abnormalities may serve as early markers and can differentiate FTD from other neurodegenerative disorders. Eye tracking could give more information about how individuals arrive at an answer, for example, by analyzing fixation patterns or search strategies.^60^ The use of instructionless tasks is promising for capturing pure emotion recognition impairments without specific task-related language strategies.^35^ Although the development of eye tracking-based cognitive measures is rapidly growing, several limitations were reported regarding sample sizes, methodological factors, or data loss due to participant-related factors.^17,44,55,60^ Such limitations are very important to be reported by all studies and taken into consideration when validating eye tracking tools in clinical cohorts.

Next to the limitations reported in the eye tracking studies, this review also identified some specific eye tracking-related gaps that need to be addressed. First, some disparities in eye tracking results have emerged between studies. A possible explanation could be the use of different methodological approaches, such as variations in eye tracking equipment, sampling rates, task instructions, or stimulus design, all of which can influence data quality and comparability. This methodological limitation underscores the importance of developing standardized protocols to enhance the reliability and generalizability of eye tracking tests. Second, existing studies do not capture the full range of major cognitive domains, leaving important aspects of neuropsychological functioning unexplored. For instance, executive functioning involves far more than anticipatory saccades, and social cognition includes abilities such as moral reasoning, Theory of Mind (ToM), and understanding of social norms.^7^ This highlights a notable gap in the current literature, as disorders in various aspects of executive functioning and social cognition are considered the hallmark of most FTD variants.^8^ Extending eye tracking paradigms to dynamic social settings, such as using videos or VR-interactions, could help identify real-time difficulties in social cognition in a more ecologically valid way.

Computer-, smartphone-, and tablet-based assessments such as CANTAB, TabCAT-EXAMINER, and Cogstate, have demonstrated the ability to detect cognitive impairment in neurodegenerative disorders, including FTD. However, these assessments were administered during supervised clinic or research visits, currently limiting their ability to capture cognitive functioning in real-world settings. Remote testing could have a lot of potential benefits, as individuals can participate from home, avoiding travel fatigue and stress. This is especially valuable for individuals living in rural areas or with mobility limitations.

Despite the growing interest in VR as a tool for cognitive assessment, research on its use in FTD remains limited. To date, only a single feasibility study has explored the use of VR in this people with FTD.^85^ In addition, the current literature does not yet provide a clear framework for how VR can be used to assess cognition. Critical questions remain unanswered regarding which cognitive functions are best evaluated through VR, how to tailor virtual tasks to impairments associated with FTD subtypes, and whether VR-based assessments offer more diagnostic value over other digital or pen-and-paper tests.

### Remote active assessment

Remote active assessments remain relatively underexplored in FTD but show considerable promise for longitudinal monitoring of cognitive functioning. Recent studies have examined smartphone-based cognitive assessments, including the ALLFTD app and the Beiwe platform. Preliminary findings suggest that these approaches are feasible and acceptable for individuals across the FTD spectrum and may be sensitive to early disease-related cognitive changes. In addition, one study by Reilly et al.^80^ demonstrated the feasibility of portable eye tracking in a home setting. Together, these findings highlight the potential of remote digital assessments to capture subtle cognitive and behavioral changes in ecologically valid environments while reducing participant burden and enabling more frequent monitoring over time. However, evidence remains limited, and further validation in larger longitudinal cohorts is needed before these technologies can be implemented for disease monitoring.

In addition to the included studies in this scoping review, other digital cognitive platforms are beginning to emerge. The work of the GENFI consortium^95^ reported normative data of the Ignite app, a tablet-based cognitive assessment tool specially addressing FTD-related symptoms, by administering the app in more than 2000 cognitive healthy adults. Most tasks showed significant moderate-to-strong correlations with established pen-and-paper tests and moderate to excellent test-retest reliability.^95^ Their work supports the feasibility of the app as it can be successfully used across a wide range of ages and provides preliminary evidence that its tests capture cognitive performance consistent with trajectories of normal aging. Although this study was outside the scope of this review as it did not include a FTD cohort, it highlights ongoing efforts toward standardization and clinical implementation of tablet-based assessments.

### Remote active assessments

Wearable technologies show clear potential for capturing real-world behavioral and physiological changes in individuals with FTD, particularly in domains such as activity levels, sleep, circadian rhythms, and apathy.^89–93^ These approaches offer important advantages over traditional assessments, including ecological validity, reduced patient burden, and the ability to capture fluctuations in behavior over time. Actigraphy appears sensitive to clinically relevant features such as disrupted rest–activity rhythms and reduced activity associated with apathy.^89,90^ However, the reported limitations highlight the need for careful interpretation and validation against gold-standard behavioral measures. In addition, more research is needed to integrate wearables with other (digital) measures to gain a comprehensive view of cognitive and behavioral changes in FTD.

Smartphone-based assessments such as the ALLFTD app and the Beiwe platform are able to collect passive measures that are associated with neurobehavioral domains such as GPS, communication logs, on-and-off cycles and battery percentages.^88,94^ These passive digital measures may provide ecologically valid markers of real-world functioning by capturing changes in daily routines, social engagement, activity levels, and behavioral patterns over time. Such approaches may be particularly valuable in FTD, where behavioral and motivational changes are prominent clinical features. However, interpretation of these passive signals remains complex, as changes may also be influenced by environmental, technical, or individual lifestyle factors.

### Assessment in presymptomatic groups

To date, only a few studies have investigated digital health technologies in presymptomatic carriers of FTD-related pathogenic variants.^55,86,87,94^ Yet, this population offers a crucial opportunity to detect subtle cognitive changes that may emerge years before clinical diagnosis. In addition, digital health technologies could enable continuous and non-invasive monitoring, which is particularly valuable in rare genetic populations that are geographically dispersed. Such approaches allow for the development of sensitive digital markers that can track disease progression and provide insights into the earliest changes of the disease. Importantly, digital health technologies show potential as outcome measures in clinical trials targeting the presymptomatic stage. Future research should therefore prioritize this population, where digital health technologies are likely to have great potential.

### Challenges for clinical implementation

Taken together, the current body of research on digital health shows initial steps toward implementation in clinical settings, while also highlighting important opportunities for further development. First, as most studies have an experimental design, many neuropsychologists are still hesitant to adopt digital methods due to insufficient scientific evidence on clinical relevance and diagnostic accuracy.^96,97^ While many studies demonstrate group differences between individuals with FTD and healthy controls or other neurodegenerative disorders, psychometric evaluation remained limited overall, such as test–retest reliability, diagnostic accuracy, or normative reference values. Similarly, most studies rely on cross-sectional designs and do not examine whether digital assessments are stable over time or sensitive to disease progression. Moreover, all eye tracking studies, and several tablet- and wearable-based assessments, were conducted using fixed, in-person setups, despite one of the key advantages of digital health technologies being their potential for remote and ecologically valid assessment. This raises an important question regarding their added clinical value in settings where individuals already undergo standard neuropsychological testing. Notably, very few studies have evaluated the incremental validity of digital assessments, such as eye tracking, over traditional cognitive measures or other biomarkers. As a result, it remains unclear whether these approaches provide additional diagnostic or prognostic information beyond established assessments when administered in-clinic. Future studies should therefore prioritize systematic validation, including larger cohorts, longitudinal designs, and formal evaluation of psychometric properties, to determine their potential role as reliable digital biomarkers in FTD.

Another important methodological consideration for future studies is the use of burst designs. This approach is particularly relevant in the preclinical stage of FTD, where the focus is on subtle changes such as a reduced learning effect across repeated assessments.^95^ By capturing these more subtle variations in performance, burst designs may provide greater sensitivity for detecting early disease-related changes, making them highly valuable in the context of preclinical FTD research.

Moreover, research on digital health technologies should be conducted in culturally diverse countries. At this moment, there is a lack of sufficiently large and diverse normative datasets, which limits the use of these tests for diagnostic purposes. However, in low- and middle-income countries, limited financial resources may significantly hinder the study and implementation of digital health technologies.^98^ Research highlights that inadequate funding, infrastructure deficits, and high costs are major barriers preventing the evaluation and integration of digital health interventions in these settings.^99^ Thus, multinational collaborations should incorporate low- and middle-income countries in order to study the feasibility of digital health technologies in diverse populations and to improve the generalizability of findings.

Lastly, decades of neuroscientific research have made it clear that no single biomarker can be used for a definitive diagnosis, and successful diagnosis relies heavily on combining multimodal approaches. By integrating multiple technologies such as eye tracking (ideally mobile variants^^100^^) and tablet- and smartphone-based tasks, clinicians could obtain a more comprehensive and ecologically valid understanding of cognitive and behavioral changes. Future research should focus on developing and validating assessments that combine these technologies in a user-friendly manner, suited for both clinical and remote environments.

## Conclusions

This scoping review highlights the expanding field of digital health technologies in FTD. Importantly, some digital health technologies are already able to differentiate FTD from other clinical groups or healthy controls, but others remain in the feasibility stage. Having established that they are feasible, the next step is examining their clinical validity and reliability, exploring whether they offer superior diagnostic value compared to traditional pen-and-paper tests. In addition, several gaps in the current literature remain, causing the need for standardized protocols, larger and diverse cohorts, and the use of burst designs in remote settings. Future research should focus on integrating digital health technologies in clinical research to ensure they are user-friendly, clinically meaningful, and capable of capturing the multifaceted nature of FTD. These are important steps that need to be addressed before implementation into clinical practice and trials can be considered.

## Supplemental material

Supplemental material - Digital health technologies in frontotemporal dementia: A scoping review Supplemental material for Digital health technologies in frontotemporal dementia: A scoping review by Liset de Boer, Olaiya A. Aro, Harro Seelaar, Lize C. Jiskoot, and Jackie M. Poos in Digital Health.

Supplemental material - Digital health technologies in frontotemporal dementia: A scoping review Supplemental material for Digital health technologies in frontotemporal dementia: A scoping review by Liset de Boer, Olaiya A. Aro, Harro Seelaar, Lize C. Jiskoot, and Jackie M. Poos in Digital Health.

## Data Availability

Data sharing is not applicable to this article as no datasets were generated or analyzed during the current study. Literature search results can be made available upon request.*
