# Supplementary material for: Digital health technologies in frontotemporal dementia: A scoping review
Source: Digit Health. 2026 Sep 15;12:20552076261483445. doi: 10.1177/20552076261483445 (PMC13583275; doi:10.1177/20552076261483445)
Supplement: Supplementary file 1 [file sj-pdf-1-dhj-10.1177_20552076261483445.pdf]

Supplementary file 1

Search strategy

**Database searched**	**Platform**	**Years of coverage**	**Records**	**Records after duplicates removed**
Medline ALL	Ovid	1946 - Present	941	935
Embase	Embase.com	1971 - Present	1368	511
Web of Science Core Collection*	Web of Knowledge	1975 - Present	1420	544
Cochrane Central Register of Controlled Trials	Wiley	1992 - Present	29	12
**Total**			**3758**	**2002**

*Science Citation Index Expanded (1975-present) ; Social Sciences Citation Index (1975-present) ; Arts & Humanities Citation Index (1975-present) ; Conference Proceedings Citation Index- Science (1990-present) ; Conference Proceedings Citation Index- Social Science & Humanities (1990-present) ; Emerging Sources Citation Index (2005-present)

No other database limits were used than those specified in the search strategies

Medline

(exp "Mobile Applications"/ OR exp "Virtual Reality"/ OR exp "Augmented Reality"/ OR exp "Artificial Intelligence"/ OR exp "Machine Learning"/ OR exp "Smart Glasses"/ OR exp "Eye tracking Technology"/ OR exp *"Eye Movements"/ OR exp "Digital Technology"/ OR (((innovative* OR new OR digital* OR computeri* OR linguis*) ADJ3 (mark* OR biomark*)) OR ehealth* OR e-health* OR electronic-health* OR mhealth* OR m-health* OR mobile-health* OR virtual*-medicine* OR ((mobile* OR tablet*) ADJ3 (app* OR phone*)) OR smartphone* OR smart-phone* OR ((remote* OR digital*) ADJ3 (care* OR health*)) OR ((virtual* OR augment*) ADJ3 (realit*)) OR genAI* OR gen-ai OR chatgpt* OR chat-gt* OR chatbot* OR chat-bot* OR ((microsoft* OR MS) ADJ6 (copilot OR co-pilot)) OR bingchat OR bing-chat OR ((artificial* OR machine* OR ambient*) ADJ3 (intelligen*)) OR ((automat*) ADJ3 (reason*)) OR ((cognitive*) ADJ3 (technol* OR robotic*)) OR ((machine* OR deep) ADJ3 (learn*)) OR ((neural*) ADJ3 (network*)) OR ((large*) ADJ3 (language*) ADJ3 (model*)) OR video* OR ((online) ADJ3 (appointment* OR meeting)) OR holo-lens* OR holograph* OR hololens* OR holo-graph* OR ((home* OR remote*) ADJ3 (monitor*)) OR wearable* OR smartwatch* OR smart-watch* OR ((audio* OR speech*) ADJ3 (record* OR sample* OR transcript*)) OR ((transcript*) ADJ3 (cookie*)) OR connect*-speech* OR ((eye* OR ocular*) ADJ3 (track* OR movement* OR moving)) OR (computer* NOT (computer*-tomograph* OR computer*-assis*))).ab,ti,kf.) **AND** ("Cognition"/ OR exp "Attention"/ OR exp " Cognitive Reserve"/ OR exp "Executive Function"/ OR exp "Learning"/ OR exp "Memory"/ OR exp "Intelligence"/ OR exp "orientation"/ OR exp "Perception"/ OR (cognit* OR attention* OR executive-funct* OR learning OR memory* OR memories OR recall OR recognition OR

repetition*-prim* OR language* OR speech OR numeric* OR numerac* OR orientation* OR ((mental*) ADJ3 (capacit* OR load*)) OR perception* OR attentional-blink* OR ((pattern*) ADJ3 (recogn*)) OR receptive-field* OR sensor*-gat* OR signal-detect* OR theory-of-mind* OR empath* OR emotion).ab,ti,kf.) **AND** (exp "Frontotemporal Dementia"/ OR exp "Corticobasal Degeneration"/ OR exp "Supranuclear Palsy, Progressive"/ OR (((frontotemp* OR fronto-temp*) ADJ3 (dement* OR degenerat*)) OR ((frontal* OR temporal*) ADJ3 (variant*)) OR ((primary) ADJ3 (progressive) ADJ3 (aphas*)) OR ((semantic*) ADJ3 (dement*)) OR ((corticobas* OR cortical-basal*) ADJ3 (degenerat* OR syndrom*)) OR ((FTD) ADJ3 (ALS)) OR ((progressive*) ADJ3 (supranucl*) ADJ3 (pals*)) OR ((ppa) ADJ3 (variant*)) OR ((PPA) ADJ1 (g OR l OR s))).ab,ti,kf.) **NOT** (news OR congres* OR abstract* OR book* OR chapter* OR dissertation abstract*).pt.

Embase

('digital biomarker'/exp OR 'mobile application'/exp OR 'virtual reality'/exp OR 'augmented reality'/exp OR 'generative model'/exp OR 'chatbot'/exp OR 'artificial intelligence'/exp OR 'artificial general intelligence'/exp OR 'generative artificial intelligence'/exp OR 'explainable artificial intelligence'/exp OR 'machine learning'/de OR 'artificial neural network'/exp OR 'feature learning (machine learning)'/exp OR 'large language model'/exp OR 'wearable sensor'/exp OR 'eye tracking'/exp OR 'eye tracking technology'/exp OR 'connected speech'/exp OR 'speech analysis'/exp/mj OR 'eye movement'/exp/mj OR 'digital technology'/exp OR (((innovative* OR new OR digital* OR computeri* OR linguis*) NEAR/3 (mark* OR biomark*)) OR ehealth* OR e-health* OR electronic-health* OR mhealth* OR m-health* OR mobile-health* OR virtual*-medicine* OR ((mobile* OR tablet*) NEAR/3 (app* OR phone*)) OR smartphone* OR smart-phone* OR ((remote* OR digital*) NEAR/3 (care* OR health*)) OR ((virtual* OR augment*) NEAR/3 (realit*)) OR genAI* OR gen-ai OR chatgpt* OR chat-gt* OR chatbot* OR chat-bot* OR ((microsoft* OR MS) NEAR/6 (copilot OR co-pilot)) OR bingchat OR bing-chat OR ((artificial* OR machine* OR ambient*) NEAR/3 (intelligen*)) OR ((automat*) NEAR/3 (reason*)) OR ((cognitive*) NEAR/3 (technol* OR robotic*)) OR ((machine* OR deep) NEAR/3 (learn*)) OR ((neural*) NEAR/3 (network*)) OR ((large*) NEAR/3 (language*) NEAR/3 (model*)) OR video* OR ((online) NEAR/3 (appointment* OR meeting)) OR holo-lens* OR holograph* OR hololens* OR holo-graph* OR ((home* OR remote*) NEAR/3 (monitor*)) OR wearable* OR smartwatch* OR smart-watch* OR ((audio* OR speech*) NEAR/3 (record* OR sample* OR transcript*)) OR ((transcript*) NEAR/3 (cookie*)) OR connect*-speech* OR ((eye* OR ocular*) NEAR/3 (track* OR movement* OR moving)) OR (computer* NOT (computer*-tomograph* OR computer*-assis*))):ab,ti,kw) **AND** ('cognition'/de OR 'attention'/exp OR 'cognitive reserve'/exp OR 'executive function'/exp OR 'learning'/exp OR 'memory'/exp OR 'mental capacity'/de OR 'intelligence'/exp OR 'mental performance'/exp OR 'numerical cognition'/exp OR 'orientation'/exp OR 'perception'/exp OR (cognit* OR attention* OR executive-funct* OR learning OR memory* OR memories OR recall OR recognition OR repetition*-prim* OR language* OR speech OR numeric* OR numerac* OR orientation* OR ((mental*) NEAR/3 (capacit* OR load*)) OR perception* OR attentional-blink* OR ((pattern*) NEAR/3 (recogn*)) OR receptive-field* OR sensor*-gat* OR signal-detect* OR theory-of-mind* OR empath* OR emotion):ab,ti,kw) **AND** ('frontotemporal dementia'/exp OR 'corticobasal degeneration'/exp OR 'progressive supranuclear palsy'/exp OR (((frontotemp* OR fronto-temp*) NEAR/3 (dement* OR degenerat*)) OR ((frontal* OR temporal*) NEAR/3 (variant*)) OR ((primary) NEAR/3 (progressive) NEAR/3 (aphas*)) OR ((semantic*) NEAR/3 (dement*)) OR

((corticobas* OR cortical-basal*) NEAR/3 (degenerat* OR syndrom*)) OR ((FTD) NEAR/3 (ALS)) OR ((progressive*) NEAR/3 (supranucl*) NEAR/3 (pals*)) OR ((ppa) NEAR/3 (variant*)) OR ((PPA) NEAR/1 (g OR l OR s))):ab,ti,kw) NOT ([Conference Abstract]/lim) NOT 'clinical trial'/it

Web of Science

TS=(((((innovative* OR new OR digital* OR computeri* OR linguis*) NEAR/2 (mark* OR biomark*)) OR ehealth* OR e-health* OR electronic-health* OR mhealth* OR m-health* OR mobile-health* OR virtual*-medicine* OR ((mobile* OR tablet*) NEAR/2 (app* OR phone*)) OR smartphone* OR smart-phone* OR ((remote* OR digital*) NEAR/2 (care* OR health*)) OR ((virtual* OR augment*) NEAR/2 (realit*)) OR genAI* OR gen-ai OR chatgpt* OR chat-gt* OR chatbot* OR chat-bot* OR ((microsoft* OR MS) NEAR/5 (copilot OR co-pilot)) OR bingchat OR bing-chat OR ((artificial* OR machine* OR ambient*) NEAR/2 (intelligen*)) OR ((automat*) NEAR/2 (reason*)) OR ((cognitive*) NEAR/2 (technol* OR robotic*)) OR ((machine* OR deep) NEAR/2 (learn*)) OR ((neural*) NEAR/2 (network*)) OR ((large*) NEAR/2 (language*) NEAR/2 (model*)) OR video* OR ((online) NEAR/2 (appointment* OR meeting)) OR holo-lens* OR holograph* OR hololens* OR holo-graph* OR ((home* OR remote*) NEAR/2 (monitor*)) OR wearable* OR smartwatch* OR smart-watch* OR ((audio* OR speech*) NEAR/2 (record* OR sample* OR transcript*)) OR ((transcript*) NEAR/2 (cookie*)) OR connect*-speech* OR ((eye* OR ocular*) NEAR/2 (track* OR movement* OR moving)) OR (computer* NOT (computer*-tomograph* OR computer*-assis*)))) **AND** ((cognit* OR attention* OR executive-funct* OR learning OR memory* OR memories OR recall OR recognition OR repetition*-prim* OR language* OR speech OR numeric* OR numerac* OR orientation* OR ((mental*) NEAR/2 (capacit* OR load*)) OR perception* OR attentional-blink* OR ((pattern*) NEAR/2 (recogn*)) OR receptive-field* OR sensor*-gat* OR signal-detect* OR theory-of-mind* OR empath* OR emotion)) **AND** ((((frontotemp* OR fronto-temp*) NEAR/2 (dement* OR degenerat*)) OR ((frontal* OR temporal*) NEAR/2 (variant*)) OR ((primary) NEAR/2 (progressive) NEAR/2 (aphas*)) OR ((semantic*) NEAR/2 (dement*)) OR ((corticobas* OR cortical-basal*) NEAR/2 (degenerat* OR syndrom*)) OR ((FTD) NEAR/2 (ALS)) OR ((progressive*) NEAR/2 (supranucl*) NEAR/2 (pals*)) OR ((ppa) NEAR/2 (variant*)) OR ((PPA) NEAR/1 (g OR l OR s))))) **NOT** DT=(Meeting Abstract OR Meeting Summary)

Cochrane CENTRAL

((((innovative* OR new OR digital* OR computeri* OR linguis*) NEAR/3 (mark* OR biomark*)) OR ehealth* OR e NEXT/1 health* OR electronic NEXT/1 health* OR mhealth* OR m NEXT/1 health* OR mobile NEXT/1 health* OR virtual* NEXT/1 medicine* OR ((mobile* OR tablet*) NEAR/3 (app* OR phone*)) OR smartphone* OR smart NEXT/1 phone* OR ((remote* OR digital*) NEAR/3 (care* OR health*)) OR ((virtual* OR augment*) NEAR/3 (realit*)) OR genAI* OR gen NEXT/1 ai OR chatgpt* OR chat NEXT/1 gt* OR chatbot* OR chat NEXT/1 bot* OR ((microsoft* OR MS) NEAR/6 (copilot OR co NEXT/1 pilot)) OR bingchat OR bing NEXT/1 chat OR ((artificial* OR machine* OR ambient*) NEAR/3 (intelligen*)) OR ((automat*) NEAR/3 (reason*)) OR ((cognitive*) NEAR/3 (technol* OR robotic*)) OR ((machine* OR deep) NEAR/3 (learn*)) OR ((neural*) NEAR/3 (network*)) OR ((large*) NEAR/3 (language*) NEAR/3 (model*)) OR video* OR ((online) NEAR/3 (appointment* OR meeting)) OR holo NEXT/1 lens* OR holograph* OR hololens* OR holo NEXT/1 graph* OR ((home* OR remote*) NEAR/3 (monitor*)) OR wearable* OR smartwatch*

OR smart NEXT/1 watch* OR ((audio* OR speech*) NEAR/3 (record* OR sample* OR transcript*)) OR ((transcript*) NEAR/3 (cookie*)) OR connect* NEXT/1 speech* OR ((eye* OR ocular*) NEAR/3 (track* OR movement* OR moving)) OR (computer* NOT (computer* NEXT/1 tomograph* OR computer* NEXT/1 assis*))):ab,ti,kw) **AND** ((cognit* OR attention* OR executive NEXT/1 funct* OR learning OR memory* OR memories OR recall OR recognition OR repetition* NEXT/1 prim* OR language* OR speech OR numeric* OR numerac* OR orientation* OR ((mental*) NEAR/3 (capacit* OR load*)) OR perception* OR attentional NEXT/1 blink* OR ((pattern*) NEAR/3 (recogn*)) OR receptive NEXT/1 field* OR sensor* NEXT/1 gat* OR signal NEXT/1 detect* OR theory NEXT/1 of NEXT/1 mind* OR empath* OR emotion):ab,ti,kw) **AND** ((((frontotemp* OR fronto NEXT/1 temp*) NEAR/3 (dement* OR degenerat*)) OR ((frontal* OR temporal*) NEAR/3 (variant*)) OR ((primary) NEAR/3 (progressive) NEAR/3 (aphas*)) OR ((semantic*) NEAR/3 (dement*)) OR ((corticobas* OR cortical NEXT/1 basal*) NEAR/3 (degenerat* OR syndrom*)) OR ((FTD) NEAR/3 (ALS)) OR ((progressive*) NEAR/3 (supranucl*) NEAR/3 (pals*)) OR ((ppa) NEAR/3 (variant*)) OR ((PPA) NEAR/1 (g OR l OR s))):ab,ti,kw) NOT ("conference abstract":kw OR Trial registry record:pt)
