# Supplementary material for: Digital health technologies in frontotemporal dementia: A scoping review
Source: Digit Health. 2026 Sep 15;12:20552076261483445. doi: 10.1177/20552076261483445 (PMC13583275; doi:10.1177/20552076261483445)
Supplement: Supplementary file 2 [file sj-pdf-2-dhj-10.1177_20552076261483445.pdf]

Supplementary Table 1. Characteristics and outcomes of included studies in the scoping review.

study	year	Population	Dementia Diagnosis	Type of digital tool	Cognitive/behavioral domains reported	Key finding	Psychometric properties/ Implementation properties	Reported issues/ limitations
IN-PERSON ACTIVE ASSESSMENT								
**Becker et al.[1]**	2019	ALS (n=119)<br>HC (n=47)	El Escorial[2]	Head-mounted video-oculography system (EyeSeeCam®)	**Eye tracking**: saccades (self-paced, delayed, anti-saccades)<br>**Cognition**: global cognitive function (ECAS)	Saccadic intrusions enlarged in ALS, associated with oculomotor and neuropsychological tests of executive functions	**Construct validity** (correlations with ALS-specific ECAS score and ECAS fluency (all *p*<0.05)	None
**Becker et al.[3]**	2023	ALS (n=94)<br>PSP (n=50)<br>HC (n=48)	El Escorial[2]<br>Höglinger[4]	Head-mounted video-oculography system (EyeSeeCam®)	**Eye tracking**: saccades, SWJ	ALS and PSP have abnormal saccades, relationship between amplitude and frequency differs between diseases	None	Probable recording noise of eyetracker
**Behler et al.[5]**	2021	C9orf72 carrier (n=22)<br>Other ALS pathogenic variants (n=28)	NA	Head-mounted video-oculography system (EyeSeeCam®)	**Eye tracking**: saccades (reactive, delayed, anti-saccades), SP<br>**Cognition**: global cognitive function (ECAS)	Impairment in executive oculomotor control in C9orf72 carriers. Anti-saccade performance correlated with ECAS	**Construct validity** (correlations with ECAS total score and executive function (*r*=-0.59-0.74))	Small sample size, did not include intensive neuropsychological testing
**Boxer et al.[6]**	2006	bvFTD (n=14)<br>svPPA (n=10)<br>nfvPPA (n=7)<br>AD (n=18)<br>HC (n=20)	Neary[7]<br>Gorno-Tempini[8]<br>McKhann[9]	Dual Purkinje Image Eye Tracker (Generation 6.1; Fourward Systems)	**Eye tracking**: saccades (pro, anti-saccades), SP<br>**Cognition**: global cognitive function (MMSE), memory, language, executive function	Patients with FTD impaired in anti-saccade task and SP, which correlated with tests for executive function and frontal gray matter volume	**Construct validity** (correlations with tests for executive function (*r*=0.53-0.70) and spatial function (*r*=0.57))<br>**Neuroanatomical validity** (correlations with gray matter volume in FEF (*r*=0.81))	None
**Boxer et al.[10]**	2012	bvFTD (n=15)<br>PSP (n=8)<br>CBD (n=4)<br>AD (n=10)<br>HC (n=27)	Neary[7]<br>Litvan[11]<br>Boeve[12]<br>McKhann[9]	Dual Purkinje Image Eye Tracker (Generation 6.1; Fourward Systems)	**Eye tracking**: saccades (pro, anti-saccades)	Impaired anti-saccade in FTD and AD. Velocity and gain most impaired in PSP. High AUC values for distinguishing PSP from FTD and AD. Correlations with dorsal midbrain volume	**Neuroanatomical validity** (correlations with dorsal mid brain volume (p= 0.03))<br>**Diagnostic accuracy** (AUC=0.40-0.99)	None
**Burrell et al.[13]**	2012	bvFTD (n=10)<br>svPPA (n=7)<br>nfvPPA (n=7)<br>HC (n=23)	Neary[7]	Saccadometer Advanced (Ober Consulting)	**Eye tracking**: saccades (reflexive, anti-saccades)	Saccades abnormal in FTD, associated with atrophy of the left frontal eye field	**Neuroanatomical validity** (correlations with frontal and cingulate eye field (*p*<0.001))	bvFTD and PPA variants taken together in analysis
**Burrell et al.[14]**	2013	ALS (n=24)<br>HC (n=119)	El Escorial[2]	Saccadometer Advanced (Ober Consulting)	**Eye tracking**: reflexive saccades, anti-saccades<br>**Behavior**: motor and functional impairment (ALSFRS-R)	Early saccades increased ALS. Did not correlate with motor or functional impairment	**Construct validity** (but no correlations with motor and functional impairment (*r*=-0.25—0.05, *p*>0.05))	Did not include intensive neuropsychological testing
**Choi et al.[15]**	2021	PSP (n=123)<br>PD (n=80)	Höglinger[4]<br>Gibb[16]	Video-Frenzel goggle system (SLMED)	**Eye tracking**: saccades, SP, SWJ<br>**Cognition**: global cognitive function (MMSE)	PSP patients showed slower vertical saccades and SP than PD	**Neuroanatomical validity** (correlations with brainstem, midbrain and pons (*r*=0.39-0.54))	Cross- sectional design, did not include intensive neuropsychological testing
**Coppe et al.[17]**	2012	bvFTD (n=8)<br>svPPA (n=2)<br>nfvPPA (n=4)<br>AD (n=11)<br>HC (n=15)	Neary[7]<br>McKhann[9]	Eyelink 1000 (SR Research, Mississauga, Ontario, Canada)	**Eye tracking**: predictive saccades, SP<br>**Cognition**: global cognitive function (MMSE), language, visuospatial function, attention, memory, executive function	Deficit of predictive acceleration in a early phase of the FTD when usual measures of SP are still normal	**Diagnostic accuracy** (sensitivity (0.85-0.86), specificity (0.86-1.00))	None

**Deravet et al.[18]**	2021	bvFTD (n=16)<br>PPA (n=6)<br>C9orf72 carrier (n=2)<br>HC (n=51)	Rascvosky[19]<br>Gorno-Tempini[8]	Eyelink 1000 (SR Research, Mississauga, Ontario, Canada)	**Eye tracking**: saccades (reactive, predictive)<br>**Cognition**: global cognitive function (MMSE)	Deficits in production of predictive saccades in FTD. Asymptomatic C9orf72 carrier showed oculomotor behavior similar to FTD patients	**Diagnostic accuracy** (sensitivity (0.86), specificity (0.96))	Small and heterogenous sample, participant excluded due to excessive data loss
**Douglass et al.[20]**	2018	bvFTD (n=20)<br>HC (n-22)	Rascovsky[19]	SMI HEDx tracker (SensoriMotoric Instruments Inc.)	**Eye tracking**: saccades (pro, predictive, self-paced), memory-guided saccades<br>**Cognition**: global cognitive function (NUCOG)	Increased latencies in bvFTD, lower number of self-paced saccades and more errors in memory-guided saccades	**Construct validity** (correlations with NUCOG (*r*=-0.45-0.71))	Small sample, possible effect of medication, did not include intensive neuropsychological testing and imaging
**Douglass et al.[21]**	2019	bvFTD (n=15)<br>HC (n=17)	Rascovsky[19]	Tobii 1750 eye tracker (Tobii, Danderyd, Sweden)	**Eye tracking**: visual search<br>**Cognition**: global cognitive function (NUCOG)	Decrease in accuracy, increase in response time and the number and length of eye movements in bvFTD	**Construct validity** (but no correlations with NUCOG)	Head movements during task, need for standardized tests
**El Haj et al.[22]**	2022	bvFTD (n=5)<br>HC (n=21)	Rascovsky[19]	Eye-tracking glasses (Pupil Lab)	**Eye tracking**: pupillometry<br>**Cognition**: global cognitive function (MMSE), (working) memory	Pupil sizes did not differ between bvFTD and HC	None	Small sample
**El Haj et al.[23]**	2024	bvFTD (n=12)<br>HC (n=12)	Rascovsky[19]	Eye-tracking glasses (Pupil Lab)	**Eye tracking**: pupillometry<br>**Cognition**: global cognitive function (MMSE), memory, language	Smaller pupil size on verbal fluency tasks and during counting in bvFTD compared to HC	None	Lack of intensive linguistic assessment and fixation condition
**Faria et al.[24]**	2018	svPPA (n=8)<br>lvPPA (n=4)<br>nfvPPA n=3)<br>HC (n=3)	Gorno-Tempini[8]	Not reported	**Eye tracking**: word picture matching<br>**Cognition**: language	svPPA less accurate and more fixations at semantically related foils. Associated with atrophy in temporal poles	**Neuroanatomical validity** (correlations with bilateral temporal poles ($\rho \approx$0.78))	Small sample
**Fieldhouse et al.[25]**	2023	bvFTD (n=18)<br>PPD (n=11)<br>HC (n=22)	Rascovsky[19]<br>DSM-V[26]	Tobii Pro X2-60 screen-based eye tracker (Tobii, Danderyd, Sweden)	**Eye tracking**: dwell time<br>**Cognition**: social cognition (FER), attention, memory, executive function, language	bvFTD patients spent less dwell time on image and on mouth area	None	Small and heterogenous sample, psychometric quality of social cognition tests is still lacking
**Garbutt et al.[27]**	2008	bvFTD (n=24)<br>svPPA (n=19)<br>nfvPPA (n=6)<br>AD (n=28)<br>CBS (n=15)<br>PSP (n=10)<br>HC (n=27)	Neary[7]<br>Boeve[12]<br>McKhann[9]<br>Litvan[11]	Dual Purkinje Image Eye Tracker (Generation 6.1; Fourward Systems)	**Eye tracking**: prosaccades, anti-saccades, SP<br>**Cognition**: global cognitive function (MMSE), memory, language, visuospatial function, executive function	All groups except svPPA impaired on anti-saccade task, bvFTD subjects able to self-correct. Eye tracking was superior to neuropsychological tests in differentiating PSP from other disorders	**Diagnostic accuracy** (AUC=0.64-1.00)<br>**Incremental validity** (comparison of oculomotor and neuropsychological variables),<br>**Neuroanatomical validity** (correlations with brain volumes (*r*=-0.43-0.05))	Majority of subjects did not have autopsy-confirmed diagnoses
**Gorges et al.[28]**	2015	ALS (n=68)<br>HC (n=31)	El Escorial[2]	Head-mounted video-oculography system (EyeSeeCam®)	**Eye tracking**: saccades (reactive, delayed, self-paced, anti-saccades), SP<br>**Cognition**: global cognitive function (ECAS)	Increased anti- and delayed saccades' errors, gaze palsy and a cerebellary type of SP disturbance in ALS	**Construct validity** (correlations with ECAS (*r*=-0.51-0.47)	Large population of participants did not show oculomotor deficits, lack of longitudinal data, did not include imaging
**Gorges et al.[29]**	2017	PSP (n=30)<br>MSA (n=18)<br>PD (n=36)<br>HC (n=23)	Höglinger[4]<br>Gilman[30]<br>Postuma[31]	EyeLink I (SR Research, Mississauga, Ontario, Canada)	**Eye tracking**: reactive saccades, SP<br>**Cognition**: global cognitive function (MMSE)	Eye movement disturbances in PSP, associated with microstructural damage in white matter tracts	**Neuroanatomical validity** (correlations with white matter in cerebral peduncle (ρ=0.81))	No post-mortem diagnosis, small sample, lack of intensive neuropsychological assessment
**Hammers et al.[32]**	2011	bvFTD (n=9)<br>AD (n =52)<br>DLB (n=10)<br>MCI (n=20)	Neary[7]<br>McKhann[9]<br>McKeith[33]<br>Petersen[34]	Cogstate battery	**Cognition**: psychomotor, attention, (working) memory, processing speed, executive function	CogState discriminates dementia groups from MCI or HC. Acceptable stability and test-retest reliability	**Reliability** (test-retest *r*=-0.19-0.80))	Small sample, only 2 time points

		HC (n=23)						
**Hammers et al.**[35]	2012	bvFTD (n=7)<br>AD (n =37)<br>DLB (n=5)<br>MCI (n=16)<br>HC (n=22)	Neary[7]<br>McKhann[9]<br>McKeith[33]<br>Petersen[34]	Cogstate battery	**Cognition**: psychomotor, attention, (working) memory, processing speed, executive function	CogState able to detect dementia, but not able to differentiate between dementia groups. Correlations with standard neuropsychological tests	**Construct validity** (correlations with Benton visual form discrimination test and visual reproduction (*r*=0.45-0.52))	Small sample, heterogenous disease stages, exclusion due to poor completion rate
**Herwig et al.**[36]	2021	PSP (n=13)<br>PD (n=15)<br>HC (n=16)	Höglinger[4]<br>Postuma[31]	HDM (HTC Vive, New Taipei City, Taiwan)	**Eye tracking**: saccade velocity and gain	Abnormalities in vertical saccade velocity and saccade gain, associated with midbrain volume	**Diagnostic accuracy** (AUC=0.95-0.96)<br>**Neuroanatomical validity** (correlations with midbrain volume in PSP (*r*=0.59-0.61))	Heterogenous disease stages, participants excluded due to insufficient oculomotor data
**Hutchings et al.**[37]	2018	bvFTD (n=20)<br>HC (n=21)	Rascovsky[19]	EyeLink 1000 (SR Research, Mississauga, Ontario, Canada)	**Eye tracking**: fixations<br>**Cognition**: social cognition (FER), attention, memory, language, visuospatial function, working memory, executive function	More fixations to the eyes of emotional faces in bvFTD compared to HC, associated with frontal and temporal areas and the cerebellum	**Neuroanatomical validity** (correlations with gray matter volume (*t*>3.89))	Use of passive viewing paradigm may not generalize to active emotion recognition tasks and may be affected by habituation
**Joshi et al.**[38]	2010	PSP (n=9)<br>HC (n=12)	Litvan[11]	Video-oculography	**Eye tracking**: fixations, SP	SP impaired in PSP	None	None
**Karpouzian-Rogers et al.**[39]	2022	svPPA (n=10)<br>non-svPPA (n=23)	Gorno-Tempini[8]	EyeLink 1000 (SR Research, Mississauga, Ontario, Canada)	**Eye tracking**: fixations<br>**Cognition**: language	On incorrectly pointed trials, participants demonstrated greater fixation duration on related foils	None	One participant excluded due to poor eye tracking trace
**Lage et al.**[40]	2021	bvFTD (n=18)<br>svPPA (n=7)<br>AD (n=18)<br>HC (n=29)	Rascovsky[19]<br>Gorno-Tempini[8]<br>McKhann[9]	OSCANN	**Eye tracking**: saccades (memory guided, prosaccades anti-saccades), SP<br>**Cognition**: global cognitive function (MMSE), memory, visuospatial function, attention, executive function	AD, bvFTD and svPPA exhibit differentiating oculomotor patterns which reflect the characteristic neuroanatomical distribution of pathology of each disease	**Diagnostic accuracy** (AUC=0.78-0.99)<br>**Construct validity** (significant correlations with neuropsychological tests (*r*=-0.99-0.90)),<br>**Neuroanatomical validity** (correlations with brain metabolism (r^2=0.59-0.72)	Small sample, patients might not be able to fully understand the most cognitively demanding tasks
**Mendez et al.**[41]	2014	bvFTD (n=5)	Rascovsky[19,42]	eMagin Z800 HMD (Virtually Better Co., Atlanta, GA)	**Behavior**: Arousal, stress, anxiety, anger, fatigue, and attention<br>**Cognition**: global cognitive function (MMSE), executive function	Patients tolerated VR without nausea or disorientation, heart rate changes, or worsening stress ratings. Responses were comparable to real world interviews	**Feasibility** (patients responded to all questions, were talkative and gave elaborate answers to avatars)	Small sample
**Merck et al.**[42]	2020	svPPA (n=9)<br>HC (n=15)	Gorno-Tempini[8]	Tobii T60 eye-tracker (Tobii, Danderyd, Sweden)	**Eye tracking**: fixations<br>**Cognition**: (working) memory, language, visuospatial function	Patients with svPPA more sensitive than HC to thematically related competitors	None	Small sample size
**Meyniel et al.**[43]	2005	bvFTD (n=23)<br>PSP (n=14)<br>HC (n=10)	Miller[44]<br>Litvan[11]	Electro-oculography	**Eye tracking**: prosaccade, anti-saccade<br>**Cognition**: global cognitive function (MMSE, FAB), social cognition<br>Behavior: apathy	bvFTD showed impaired reflexive saccade inhibition, similar to that in PSP, and a decreased ability to withhold an antisaccade	**Construct validity** (correlations with frontal lobe tests (*r*=-0.86-0.69))	None
**Nelson et al.**[45]	2023	svPPA (n=9)<br>lvPPA (n=12)<br>HC (n=26)	Gorno-Tempini[8]	EyeLink 1000 (SR Research, Mississauga, Ontario,	**Eye tracking**: taxonomic capture<br>**Cognition**: language, visuospatial function, working memory	svPPA more fixations on foils, more subject to taxonomic blurring	None	Working-memory and object recognition impairments as potential confounds

				Canada)				
**Otero-Millan et al.[46]**	2011	PSP (n=10)<br>HC (n=14)	Litvan[11]	Scleral search-coil system), EyeLink II (SR Research, Mississauga, Ontario, Canada)	**Eye tracking**: saccades, SWJs	SWJs are larger and more frequent in PSP	**Diagnostic accuracy** (AUC=0.70-0.99)	Video-tracking techniques are generally less precise and accurate than the search coil technique
**Pinnock et al.[47]**	2009	PSP (n=7)<br>PD (n=28)<br>MSA (n=9)<br>HC (n=50)	Litvan[11]<br>Postuma[31]	Infrared limbus eye tracker (Skalar medical 6500)	**Eye tracking**: fixations, saccades	PSP shows largest and most frequent saccadic intrusions and the largest fixation displacements	None	None
**Polet et al.[48]**	2022	bvFTD (n=9)<br>AD (n=23)<br>PD (n=20)	Rascovsky[19]<br>Dubois[49]<br>Postuma[31]	Mobile EyeBrainT2	**Eye tracking**: fixations<br>**Cognition**: social cognition (FER), visuospatial function, language<br>**Behavior**: NPI	FER impairment in bvFTD was related to increase in fixations and more gaze toward the lower part of one's face	**Construct validity** (correlations with NPI (τ=-0.45-0.33)	Small sample, ophthalmological problems excluded, social cognition test not ecological valid
**Primativo et al.[50]**	2017	bvFTD (n=12)<br>svPPA (n=6)	Rascovsky[19]<br>Gorno-Tempini[8]	Eyelink II (SR Research, Mississauga, Ontario, Canada)	**Eye tracking**: anticipatory saccades<br>**Cognition**: global cognitive function (MMSE), executive function, attention, language	bvFTD but not SD patients more anticipatory saccade errors than HC, associated with frontal brain regions	**Construct validity** (correlations with neuropsychological tests (r^2=-0.60-0.46))<br>**Neuroanatomical validity** (correlations with FEF, t=-0.03-0.03)	Small sample, machine learning results are not as interpretable compared to traditional eye tracking measures
**Proudfoot et al.[51]**	2015	ALS (n=61)<br>PLS (n=7)<br>HC (n=39)	El Escorial[2]	EyeLink 1000 (SR Research, Mississauga, Ontario, Canada)	**Eye tracking**: prosaccades, anti-saccades, executive function (TMT), visual search<br>**Cognition**: global cognitive function (ACE), executive function	ALS patients were impaired on executive and visual search tasks despite normal basic saccadic function	**Feasibility** (task-completion 91-97%),<br>**Neuroanatomical validity** (correlations with functional connectivity (p<0.0031))	Medications or respiratory insufficiency as confounds, possible selection bias, one outlier control
**Rahman et al.[52]**	1999	bvFTD (n=8)<br>HC (n=8)	Gregory and Hodges[53]	CANTAB battery	**Cognition**: (working) memory, attentional set shifting, executive function, decision making	bvFTD in mild stages demonstrates cognitive deficits not usually identified using traditional tests	None	None
**Riek et al.[54]**	2023	bvFTD (n=14)<br>nfvPPA (n=5)<br>svPPA (n=3)<br>CBS (n=2)<br>PSP (n=9)<br>ALS (n=35)<br>AD (n=26)<br>PD (n=106)<br>CVD (n=123)<br>HC (n=149)	Rascovsky[19]<br>Litvan[11]<br>El Escorial[2]<br>Gorno-Tempini[8]	EyeLink 1000 (SR Research, Mississauga, Ontario, Canada)	**Eye tracking**: prosaccades, anti-saccades<br>**Cognition**: attention/working memory, executive function, language, memory, and visuospatial function	Saccades impaired in patient groups and anti-saccade parameters correlate with executive function, attention, and working memory	**Construct validity** (correlations with neuropsychological test (r=-0.35-0.21), factor analysis)	Participants excluded if they had visual field defects or poor data
**Rivaud-Pechoux et al.[55]**	2007	PSP (n=12)<br>CBS (n=8)<br>PD (n=15)<br>HC (n=10)	Litvan[11]	Electro-oculography	**Eye tracking**: prosaccades, anti-saccaddes	Normal anti-saccade error rates in PD and CBS patients and increased error rates in PSP patients	None	None
**Russell et al.[56]**	2021	bvFTD (n=19)<br>HC (n=22)	Rascovsky[19]	EyeLink 1000 (SR Research, Mississauga, Ontario, Canada)	**Eye tracking**: fixations, prosaccades, anti-saccades, SP<br>**Cognition**: global cognitive function (MMSE), working memory, attention, executive function, language	bvFTD group impaired fixation stability and anti-saccade task, correlated with frontal and parietal cortices and striatum	**Construct validity** (correlated with executive function tasks (r=-0.44-0.54))<br>**Neuroanatomical validity**	Limited number of trials, small sample size, high costs, need for longitudinal follow-up and presymptomatic studies

							(correlations with gray matter volume (r=-0.48-0.42)	
**Russell et al.**[57]	2021	bvFTD (n=18)<br>HC (n=22)	Rascovsky[19]	EyeLink 1000 (SR Research, Mississauga, Ontario, Canada)	**Eye tracking**: dwell time (FER)<br>**Cognition**: social cognition, global cognitive function (MMSE), working memory, attention, language, executive function	Less dwell time change during social cognition task in bvFTD group, associated with frontal cortices	**Construct validity** (correlated with neuropsychological tests (ρ=-0.42-0.38)), **Neuroanatomical validity** (correlations with orbitofrontal and ventromedial prefrontal cortex (ρ=0.33))	Small sample size, executive function could have impact on task, possible issues comprehending stimuli
**Sanderson Cimino et al.**[58]	2025	bvFTD (n=167)<br>PSP (n=37)<br>CBS (n=29)<br>lvPPA (n=28)<br>nfvPPA (n=44)<br>svPPA (n=49)<br>AD (n=115)<br>MCI (n=237)	Rascovsky[19]<br>Dubois[49]<br>Gorno-Tempini[8]<br>Litvan[11]<br>Petersen[34]	TabCAT-EXAMINER	**Cognition**: executive function, working memory, processing speed, memory, language, visuospatial function	Scores were normally distributed and were associated with tests of executive function, demographics, disease severity, and diagnosis	**Construct validity** (correlations with neuropsychological tests (β=0.13-0.52), factor analysis), test-retest reliability (α=0.86))	Heterogenous sample, not culturally diverse
**Seckin et al.**[59]	2016	svPPA (n=9)<br>PPA other (n=6)<br>HC (n=14)	Gorno-Tempini[8]	EyeLink 1000 (SR Research, Mississauga, Ontario, Canada)	**Eye tracking**: saccades, fixation<br>**Cognition**: language, attention, executive function, visuospatial function	svPPA showed heightened capture even on trials where they ultimately pointed to the correct target	None	None
**Shdo et al.**[60]	2022	bvFTD (n=10)<br>svPPA (n=7)	Rascovsky[19]<br>Gorno-Tempini[8]	Applied Sciences Laboratories head-mounted monocular eye tracking system	**Eye tracking**: dwell time (FER)<br>**Cognition**: social cognition, emotional valence perception, global cognitive function (MMSE)	Individuals with FTD spent less time looking at emotional faces than HC	**Construct validity** (but no correlation with MMSE)	Small sample size
**Singleton et al.**[61]	2023	bvFTD (n=14)<br>bvAD (n=12)<br>AD (n=12)<br>SCD (n=13)	Rascovsky[19]	Tobii Pro X2-60 screen-based eye tracker (Tobii, Danderyd, Sweden)	**Eye tracking**: dwell time (FER)<br>**Cognition**: social cognition, global cognitive function (MMSE), attention, memory, language, executive function	bvAD showed a similar eye-tracking signature to patients with bvFTD	None	Small and heterogenous sample, used dwell time instead of (not in line with previous studies), only studied mouth and nose area
**Terao et al.**[62]	2013	PSP (n=36)<br>PD (n=66)<br>HC (n=58)	Litvan[11]	Electro-oculography	**Eye tracking**: saccades (visually- and memory-guided)	Vertical and horizontal saccades were affected in PSP	None	Small sample size, noise in records, EOG does not allow fully accurate assessments
**Ungrady et al.**[63]	2019	svPPA (n=7)<br>lvPPA (n=2)<br>AD (n=1)	Gorno-Tempini[8]	SMI HEDx tracker (SensoriMotoric Instruments Inc.)	**Eye tracking**: fixations, saccades<br>**Cognition**: language, global cognitive function (MoCA), attention, executive function	Patients with anomia show a lower fixation count, higher saccade count, and slower saccade velocity for items that were named incorrectly	**Construct validity** (correlations with neuropsychological tests (r=-0.35-0.48))	No correlation with language measures, cross-sectional, did not rule out oculomotor difficulties
**Viskontas et al.**[64]	2011	bvFTD (n=12)<br>svPPA (n=6)<br>AD (n=17)<br>HC (n=21)	Neary[7]<br>McKhann[9]	Applied Science Laboratories infrared eye tracker (model 504HS)	**Eye tracking**: visual search<br>**Cognition**: global cognitive function (MMSE), language, visuospatial function, memory, attention, executive function	BvFTD have less accurate performance only on the most difficult search task. svPPA were as accurate as HC. Correlation with gray matter volume in dorsal frontoparietal network	**Neuroanatomical validity** (correlations with gray matter volume (max T=5.07-6.74))	Ceiling effects in HC and svPPA, Participants who were colorblind or who had eye surgery were excluded

**Walenski et al.**[65]	2021	nfvPPA (n=10)<br>lvPPA (n=10)<br>HC (n=15)	Gorno-Tempini[8]	Applied Science Laboratories (ASL) 6000 remote eye-tracker	**Eye tracking**: fixations<br>**Cognition**: language, global cognitive function (MMSE), working memory	Delayed thematic prediction in both nfvPPA and lvPPA and abnormal thematic integration only in nfvPPA	None	Small sample sizes
**Wang et al.**[66]	2026	svPPA (n=6)<br>nfvPPA (n=5)<br>lvPPA (n=10)	Gorno-Tempini[8]	Applied Science Laboratories (ASL) 6000 remote eyetracker	**Eye tracking**: fixations<br>**Cognition**: language	Impairments in comprehension of past time reference for nfvPPA and lvPPA	None	Small sample sizes, no independent measure of executive function
**Watanabe et al.**[67]	2023	PSP (n=19)<br>PD (n=29)<br>SCD (n=21)<br>HC (n=19)	Höglinger[4]<br>Postuma[31]<br>Gilman[30]	Tobii Pro Nano and PCEye Mini (Tobii, Danderyd, Sweden)	**Eye tracking:** fixations, saccades (reading)<br>**Cognition**: global cognitive function (MoCA)	Reading velocity and fixation/saccade ratio altered in PD. Vertical gaze parameters showed aberrant values in PSP	**Construct validity** (correlations with MoCA (ρ=-0.61-0.49)),<br>**Diagnostic accuracy** (sensitivity (92.8-95.5%), specificity (64.8-78.9%)	Heterogenous sample
**Wright et al.**[68]	2022	PSP (n=11)<br>HC (n=20)	Höglinger[4]	EyeBrain T2® video eye tracker	**Eye tracking**: saccades	In PSP, accuracy is lower across all saccades compared to HC, but velocity is lower only for reflexive saccades	None	Small sample size, did not include PD in analysis, possible effect of fatigue
**Zaino et al.**[69]	2023	ALS (n=18)<br>HC (n=13)	El Escorial[2]	Applied Science Laboratories (ASL) 504 eyetracker	**Eye tracking**: saccades (visually guided, memory-guided, anti-saccades), visual search<br>**Cognition**: global cognitive function (ECAS), memory, visuospatial function, language, executive function	Separate profiles of visually guided saccades between spinal (short saccades) and bulbar (slow saccades) ALS	**Construct validity** (correlations with ECAS (ρ=-0.64—0.52))<br>**Neuroanatomical validity** (correlations with gray matter volume (ρ=-0.94-0.94)	Individuals who were unable to maintain sitting position were excluded
REMOTE ACTIVE ASSESSMENT								
**Berry et al.**[70]	2019	ALS (n=23)	El Escorial[2]	Beiwe battery	**Cognition**: language<br>Behavior: GPS, accelerometer, on-and-off-cycle, communication logs	Smartphone-based self-reports showed high agreement with in-clinic measures, and enabled frequent monitoring that detected progressive changes over time	**Construct validity** (correlations with clinic-based self-report (r=0.93))	None
**Reilly et al.**[71]	2022	svPPA (n=7)<br>lvPPA (n=2)<br>AD (n=1)	Gorno-Tempini[8]	SMI HEDx tracker (SensoriMotoric Instruments Inc.)	**Eye tracking**: fixations, dwell time<br>Cognition: global cognitive function (MoCA), language, memory	More visual fixations and greater visual fixation dispersion predictive of expressive vocabulary dropout in PPA	None	Small and heterogenous sample, lack of precision of experimental control
**Taylor et al.**[72]	2023	bvFTD (n=24)<br>PSP (n=5)<br>svPPA (n=6)<br>nfvPPA (n=4)<br>lvPPA (n=1)<br>FTD-ALS (n=2)<br>CBS (n=4)<br>Presymptomatic (n=89)<br>Prodromal (n=45)	Rascovsky[19]<br>Höglinger[4]<br>Gorno-Tempini[8]<br>El Escorial[2]<br>Armstrong[73]	ALLFTD battery	**Cognition**: executive function, language, memory, motor function<br>**Behavior**: screen time, step count, app usage, GPD, battery life	ALLFTD app is feasible and well accepted in FTD	**Feasibility** (adherence=59-78.4%)<br>**Acceptability** (93.5% found task instructions clear, 89.4% found comfortable to read, 97.6% found completion time acceptable)	Participants excluded due to software error, limited ethnocultural, socioeconomic, and language diversity
**Staffaroni et al.**[74]	2024	bvFTD (n=36)<br>PSP (n=6)<br>svPPA (n=10)<br>nfvPPA (n=4)<br>lvPPA (n=2)<br>FTD-ALS (n=2)	Rascovsky[19]<br>Höglinger[4]<br>Gorno-Tempini [8]<br>El Escorial [2]<br>Armstrong [73]	ALLFTD battery	**Cognition**: global cognitive function (MoCA), executive function, language, memory, motor function	Smartphones could offer a feasible, reliable, valid, and scalable solution for remote evaluations of FTD and may improve early detection	**Construct validity** (correlations with neuropsychological tests (r=0.40-0.66)<br>**Neuroanatomical validity** (correlations with	Floor effects in FTD, limited ethnocultural, socioeconomic, and language diversity, need for longitudinal analyses

		ALS (n=1)<br>AD (n=1)<br>CBS (n=5)<br>Presymptomatic (n=195)<br>Prodromal (n=66)					frontoparietal and subcortical volumes (β=0.34-0.50))<br>**Diagnostic accuracy** (AUC=0.87-0.93)<br>**Feasibility** (adherence 74.5-82.5%)<br>**Internal consistency** (α=0.84-0.99)<br>**Test-retest reliability** (ICC= 0.77-0.95)	
**REMOTE PASSIVE ASSESSMENT**								
**Anderson et al.[75]**	2009	bvFTD (n=13)<br>AD (n=5)<br>HC (n=11)	Neary[7]<br>McKhann[9]	Actiwatch (Cambridge Neurotechnology, UK)	**Behavior**: sleep, activity/rest cycle<br>**Cognition**: global cognitive function (MMSE)	Sleep-wake disturbances in FTD	None	Small sample size, non-wear in 2 participants
**Filardi et al.[76]**	2024	bvFTD (n=27)<br>HC (n=24)	Rascovsky[19]	Micro Motionlogger Actiwatch Watch (Ardsley, NY)	**Behavior**: sleep, activity/rest cycle	Patients with bvFTD exhibit longer sleep duration and lower sleep quality	**Neuroanatomical validity** (correlations with cortical thickness (p<0.05–0.001))	Sleep diaries of 3 participants were incomplete and excluded, cross-sectional, small sample size
**Merrillees et al.[77]**	2013	bvFTD (n=13)<br>svPPA (n=9)<br>Caregivers (n=22)	Neary[7]	MiniMitter Actiwatch monitor	**Behavior**: physical mobility, NPI, sleep<br>**Cognition**: global cognitive function (MMSE)	Lower levels of activity in bvFTD and apathy correlated with caregiver emotional distress	**Construct validity** (correlations with NPI (ρ=0.57-0.76)	Small sample size, no control data, limited assessment of apathy and depression
**Nagels et al.[78]**	2006	bvFTD(n=9)<br>AD (n=65)<br>DLB (n=16)<br>MXD (n=20)	Neary[7]<br>McKhann[9]<br>McKieth[33]	Motionlogger (Ambulatory monitoring, New York, USA)	**Behavior**: activity levels, agitation<br>**Cognition**: global cognitive function (MMSE)	Patients with higher agitation and lower MMSE had higher levels of activity during the day	**Construct validity** (correlations with behavioral measure and MMSE (r=0.19-0.35))	Did not include control group
**Paolilo et al.[79]**	2024	bvFTD (n=25)<br>PSP (n=4)<br>svPPA (n=6)<br>nfvPPA (n=3)<br>lvPPA (n=1)<br>FTD-ALS (n=1)<br>CBS (n=2)<br>Presymptomatic (n=139)<br>Prodromal (n=49)	Rascovsky[19]<br>Höglinger[4]<br>Gorno-Tempini[8]<br>Armstrong[73]	ALLFTD battery	**Behavior**: smartphone use, NPI<br>**Cognition**: executive function, memory, language, visuospatial function	Prodromal and symptomatic FTD had less change in battery percentage throughout the day (a proxy for less smartphone use). Associated with worse cognitive performance and smaller brain volumes	**Construct validity** (correlations with neuropsychological tests (β=-0.65-0.38)),<br>**Neuroanatomical validity** (correlations with gray matter volume (β=-0.73-0.45)	Possible impact of hardware, software and service connection, need for analyses over longer period of time, limited demographic diversity
**Tamburrino et al.[80]**	2025	bvFTD (n=18)	Rascovsky[19]	Micro Motionlogger® Watch (Ambulatory Monitoring, Inc., Ardsley, NY), Embletta® MPR Sleep System	**Behavior**: sleep, activity/rest cycle	Actigraphy significantly overestimates total sleep time, sleep latency, and sleep efficiency compared to polysomnography	**Accuracy** (sensitivity (93-96%), specificity (57-62%))	Small sample, participants excluded due to equipment malfunction

Abbreviations: AD: Alzheimer's disease; ALS: amyotrophic lateral sclerosis; AUC: area under the curve; bvFTD: behavioral variant frontotemporal dementia; C9orf72: chromosome 9 open reading frame 72; CBS/CBD: corticobasal syndrome/degeneration; CVD: cardiovascular disease; DLB: dementia with Lewy bodies; DSM: Diagnostic and Statistical Manual of Mental Disorders; DTI: diffusion tensor imaging; ECAS: Edinburgh Cognitive and Behavioural ALS Screen; FAB: Frontal Assessment Battery; FEF: frontal eye field; FER: facial emotion recognition; HC: healthy controls; lvPPA: logopenic variant primary progressive aphasia; MMSE: Mini-Mental State Examination; MoCA: Montreal Cognitive Assessment; MSA: multiple system atrophy; MXD: mixed dementia; nfvPPA: non-fluent variant primary progressive aphasia; PD: Parkinson's disease; PDD: primary psychiatric disorder; PLS: primary lateral sclerosis; PSP: progressive supranuclear palsy; ROC: receiver operating characteristic; SCD: subjective cognitive disorder; SP: SP; svPPA: semantic variant primary progressive aphasia; SWJ: square wave jerks; TMT: Trail Making Test

REFERENCES

1. Becker W, Gorges M, Lulé D, et al. Saccadic intrusions in amyotrophic lateral sclerosis (ALS). *J Eye Mov Res* 2019; 12: 1–17.
2. Brooks BR, Miller RG, Swash M, et al. El Escorial revisited: Revised criteria for the diagnosis of amyotrophic lateral sclerosis. *Amyotrophic Lateral Sclerosis* 2000; 1: 293–299.
3. Becker W, Behler A, Vintonyak O, et al. Patterns of small involuntary fixation saccades (SIFSs) in different neurodegenerative diseases: the role of noise. *Exp Brain Res* 2023; 241: 1821–1833.
4. Höglinger GU, Respondek G, Stamelou M, et al. Clinical Diagnosis of Progressive Supranuclear Palsy: The Movement Disorder Society Criteria. *Mov Disord* 2017; 32: 853.
5. Behler A, Knehr A, Finsel J, et al. Eye movement alterations in presymptomatic C9orf72 expansion gene carriers. *J Neurol* 2021; 268: 3390–3399.
6. Boxer AL, Garbutt S, Rankin KP, et al. Medial versus lateral frontal lobe contributions to voluntary saccade control as revealed by the study of patients with frontal lobe degeneration. *J Neurosci* 2006; 26: 6354–6363.
7. Neary D, Snowden JS, Gustafson L, et al. Frontotemporal lobar degeneration: A consensus on clinical diagnostic criteria. *Neurology* 1998; 51: 1546–1554.
8. Gorno-Tempini ML, Hillis AE, Weintraub S, et al. Classification of primary progressive aphasia and its variants. *Neurology* 2011; 76: 1006–1014.
9. McKhann G, Drachman D, Folstein M, et al. Clinical diagnosis of alzheimer's disease: Report of the NINCDS-ADRDA work group⋆ under the auspices of department of health and human services task force on alzheimer's disease. *Neurology* 1984; 34: 939–944.
10. Boxer AL, Garbutt S, Seeley WW, et al. Saccade abnormalities in autopsy-confirmed frontotemporal lobar degeneration and alzheimer disease. *Arch Neurol* 2012; 69: 509–517.
11. Litvan I, Agid Y, Calne D, et al. Clinical research criteria for the diagnosis of progressive supranuclear palsy (Steele-Richardson-Olszewski syndrome). *Neurology* 1996; 47: 1–9.
12. Boeve BF, Lang AE, Litvan I. Corticobasal degeneration and its relationship to progressive supranuclear palsy and frontotemporal dementia. *Ann Neurol* 2003; 54: S15–S19.
13. Burrell JR, Hornberger FM, Carpenter RHS, et al. *Saccadic abnormalities in frontotemporal dementia*, www.cudos.ac.uk/spic.html (2012).
14. Burrell JR, Carpenter RHS, Hodges JR, et al. Early saccades in amyotrophic lateral sclerosis. *Amyotroph Lateral Scler Frontotemporal Degener* 2013; 14: 294–301.
15. Choi JH, Kim H, Shin JH, et al. Eye movements and association with regional brain atrophy in clinical subtypes of progressive supranuclear palsy. *J Neurol* 2021; 268: 967–977.
16. Gibb WRG, Lees AJ. The relevance of the Lewy body to the pathogenesis of idiopathic Parkinson's disease. *J Neurol Neurosurg Psychiatry* 1988; 51: 745–752.
17. Coppe S, Orban De Xivry J-J, Yüksel D, et al. Dramatic impairment of prediction due to frontal lobe degeneration. *J Neurophysiol* 2012; 108: 2957–2966.
18. Deravet N, Orban de Xivry JJ, Ivanoiu A, et al. Frontotemporal dementia patients exhibit deficits in predictive saccades. *J Comput Neurosci* 2021; 49: 357–369.
19. Rascovsky K, Hodges JR, Knopman D, et al. Sensitivity of revised diagnostic criteria for the behavioural variant of frontotemporal dementia. *Brain* 2011; 134: 2456–2477.
20. Douglass A, Walterfang M, Velakoulis D, et al. Behavioral variant frontotemporal dementia performance on a range of saccadic tasks. *Journal of Alzheimer's Disease* 2018; 65: 231–242.
21. Douglass A, Walterfang M, Velakoulis D, et al. Visual Search in Behavioral Variant Frontotemporal Dementia. *Journal of Alzheimer's Disease* 2019; 72: 1303–1312.
22. El Haj M, Kapogiannis D, Boutoleau-Bretonnière C. Pupillometry as an index for cognitive processing in behavioral variant FrontoTemporal Dementia: a series of case studies. *Neurocase* 2022; 28: 270–275.
23. El Haj M, Kapogiannis D, Boutoleau-Bretonnière C. Pupil size shows diminished increases on verbal fluency tasks in patients with behavioral-variant-frontotemporal dementia. *J Neurolinguistics*; 69. Epub ahead of print 1 February 2024. DOI: 10.1016/j.jneuroling.2023.101164.
24. Faria A V., Race D, Kim K, et al. The eyes reveal uncertainty about object distinctions in semantic variant primary progressive aphasia. *Cortex* 2018; 103: 372–381.
25. Fieldhouse JLP, Singleton EH, van Engelen MPE, et al. Decreased emotion recognition and reduced focus on facial hallmarks in behavioral variant frontotemporal dementia compared to primary psychiatric disorders and controls. *Eur J Neurol* 2023; 30: 2222–2229.

26. American Psychiatric Association. Diagnostic and Statistical Manual of Mental Disorders. *Diagnostic and Statistical Manual of Mental Disorders*. Epub ahead of print 22 May 2013. DOI: 10.1176/APPI.BOOKS.9780890425596.
27. Garbutt S, Matlin A, Hellmuth J, et al. Oculomotor function in frontotemporal lobar degeneration, related disorders and Alzheimer's disease. *Brain* 2008; 131: 1268–1281.
28. Gorges M, Müller HP, Lulé D, et al. Eye movement deficits are consistent with a staging model of pTDP-43 pathology in amyotrophic lateral sclerosis. *PLoS One*; 10. Epub ahead of print 1 November 2015. DOI: 10.1371/journal.pone.0142546.
29. Gorges M, Maier MN, Rosskopf J, et al. Regional microstructural damage and patterns of eye movement impairment: a DTI and video-oculography study in neurodegenerative parkinsonian syndromes. *J Neurol* 2017; 264: 1919–1928.
30. Gilman S, Wenning GK, Low PA, et al. Second consensus statement on the diagnosis of multiple system atrophy. *Neurology* 2008; 71: 670–676.
31. Postuma RB, Berg D, Stern M, et al. MDS clinical diagnostic criteria for Parkinson's disease. *Movement Disorders* 2015; 30: 1591–1601.
32. Hammers D, Spurgeon E, Ryan K, et al. Reliability of repeated cognitive assessment of dementia using a brief computerized battery. *Am J Alzheimers Dis Other Demen* 2011; 26: 326–333.
33. Mckeith IG, Sci M, Boeve BF, et al. Diagnosis and management of dementia with Lewy bodies Fourth consensus report of the DLB Consortium.
34. Petersen RC. Mild cognitive impairment as a diagnostic entity. *J Intern Med* 2004; 256: 183–194.
35. Hammers D, Spurgeon E, Ryan K, et al. Validity of a brief computerized cognitive screening test in dementia. *J Geriatr Psychiatry Neurol* 2012; 25: 89–99.
36. Herwig A, Agic A, Huppertz HJ, et al. Differentiating Progressive Supranuclear Palsy and Parkinson's Disease With Head-Mounted Displays. *Front Neurol*; 12. Epub ahead of print 23 December 2021. DOI: 10.3389/fneur.2021.791366.
37. Hutchings R, Palermo R, Bruggemann J, et al. Looking but not seeing: Increased eye fixations in behavioural-variant frontotemporal dementia. *Cortex* 2018; 103: 71–81.
38. Joshi AC, Riley DE, Mustari MJ, et al. Selective defects of visual tracking in progressive supranuclear palsy (PSP): Implications for mechanisms of motion vision. *Vision Res* 2010; 50: 761–771.
39. Karpouzian-Rogers T, Hurley R, Seckin M, et al. Eye movements as a measure of word comprehension deficits in primary progressive aphasia. *Brain Lang*; 232. Epub ahead of print 1 September 2022. DOI: 10.1016/j.bandl.2022.105165.
40. Lage C, López-García S, Bejanin A, et al. Distinctive Oculomotor Behaviors in Alzheimer's Disease and Frontotemporal Dementia. *Front Aging Neurosci*; 12. Epub ahead of print 4 February 2021. DOI: 10.3389/fnagi.2020.603790.
41. Mendez MF, Joshi A, Jimenez E. Virtual reality for the assessment of frontotemporal dementia, a feasibility study. *Disabil Rehabil Assist Technol* 2015; 10: 160–164.
42. Merck C, Noël A, Jamet E, et al. on Thematic Knowledge in Semantic Dementia: Evidence From an Eye-Tracking Paradigm. *Neuropsychology*; 34. Epub ahead of print 2020. DOI: 10.1037/neu0000616ï.
43. Meyniel C, Rivaud-Péchoux S, Damier P, et al. Saccade impairments in patients with fronto-temporal dementia. *J Neurol Neurosurg Psychiatry* 2005; 76: 1581–1584.
44. Miller BL, Ikonte C, Ponton M, et al. A study of the Lund-Manchester research criteria for frontotemporal dementia: clinical and single-photon emission CT correlations. *Neurology* 1997; 48: 937–942.
45. Nelson MJ, Moeller S, Seckin M, et al. The eyes speak when the mouth cannot: Using eye movements to interpret omissions in primary progressive aphasia. *Neuropsychologia*; 184. Epub ahead of print 6 June 2023. DOI: 10.1016/j.neuropsychologia.2023.108530.
46. Otero-Millan J, Serra A, Leigh RJ, et al. Distinctive features of saccadic intrusions and microsaccades in progressive supranuclear palsy. *Journal of Neuroscience* 2011; 31: 4379–4387.
47. Pinnock RA, McGivern RC, Forbes R, et al. An exploration of ocular fixation in Parkinson's disease, multiple system atrophy and progressive supranuclear palsy. *J Neurol* 2010; 257: 533–539.
48. Polet K, Hesse S, Morisot A, et al. *Eye-gaze Strategies During Facial Emotion Recognition in Neurodegenerative Diseases and Links With Neuropsychiatric Disorders*, www.cogbehavneurol.com (2022).
49. Dubois B, Villain N, Frisoni GB, et al. Clinical diagnosis of Alzheimer's disease: recommendations of the International Working Group. *Lancet Neurol* 2021; 20: 484–496.
50. Primativo S, Clark C, Yong KXX, et al. Eyetracking metrics reveal impaired spatial anticipation in behavioural variant frontotemporal dementia. *Neuropsychologia* 2017; 106: 328–340.

51. Proudfoot M, Menke RAL, Sharma R, et al. Eye-tracking in amyotrophic lateral sclerosis: A longitudinal study of saccadic and cognitive tasks. *Amyotroph Lateral Scler Frontotemporal Degener* 2016; 17: 101–111.
52. Rahman S, Sahakian BJ, Hodges JR, et al. Specific cognitive deficits in mild frontal variant frontotemporal dementia. *Brain* 1999; 122 (Pt 8): 1469–1493.
53. Gregory C, Neurology JH-C and B, 1996 undefined. Frontotemporal dementia: use of consensus criteria and prevalence of psychiatric features. *journals.lww.comCA Gregory, JR HodgesCognitive and Behavioral Neurology, 1996•journals.lww.com*, https://journals.lww.com/cogbehavneurol/abstract/1996/07000/Frontotemporal_Dementia__Use_of_Consensus_Criteria.1.aspx (accessed 6 April 2026).
54. Riek HC, Brien DC, Coe BC, et al. Cognitive correlates of antisaccade behaviour across multiple neurodegenerative diseases. *Brain Commun*; 5. Epub ahead of print 2023. DOI: 10.1093/braincomms/fcad049.
55. Rivaud-Péchoux S, Vidailhet M, Brandel JP, et al. Mixing pro- and antisaccades in patients with parkinsonian syndromes. *Brain* 2007; 130: 256–264.
56. Russell LL, Greaves C V., Convery RS, et al. Eye movements in frontotemporal dementia: Abnormalities of fixation, saccades and anti-saccades. *Alzheimer's and Dementia: Translational Research and Clinical Interventions*; 7. Epub ahead of print 2021. DOI: 10.1002/trc2.12218.
57. Russell LL, Greaves C V., Convery RS, et al. Novel instructionless eye tracking tasks identify emotion recognition deficits in frontotemporal dementia. *Alzheimers Res Ther*; 13. Epub ahead of print 1 December 2021. DOI: 10.1186/s13195-021-00775-x.
58. Sanderson-Cimino M, Possin KL, Mungas DM, et al. Development and validation of the TabCAT-EXAMINER: A tablet-based executive functioning battery for research and clinical trials. *Journal of the International Neuropsychological Society* 2025; 31: 242–253.
59. Seckin M, Mesulam MM, Voss JL, et al. Am I looking at a cat or a dog? Gaze in the semantic variant of primary progressive aphasia is subject to excessive taxonomic capture. *J Neurolinguistics* 2016; 37: 68–81.
60. Shdo SM, Brown CL, Yuan J, et al. Diminished Visual Attention to Emotional Faces Is Associated with Poor Emotional Valence Perception in Frontotemporal Dementia. *Dement Geriatr Cogn Disord* 2022; 51: 331–339.
61. Singleton EH, Fieldhouse JLP, van't Hooft JJ, et al. Social cognition deficits and biometric signatures in the behavioural variant of Alzheimer's disease. *Brain* 2023; 146: 2163–2174.
62. Terao Y, Fukuda H, Shirota Y, et al. Deterioration of horizontal saccades in progressive supranuclear palsy. *Clinical Neurophysiology* 2013; 124: 354–363.
63. Ungrady MB, Flurie M, Zuckerman BM, et al. Naming and Knowing Revisited: Eyetracking Correlates of Anomia in Progressive Aphasia. *Front Hum Neurosci*; 13. Epub ahead of print 11 October 2019. DOI: 10.3389/fnhum.2019.00354.
64. Viskontas I V., Boxer AL, Fesenko J, et al. Visual search patterns in semantic dementia show paradoxical facilitation of binding processes. *Neuropsychologia* 2011; 49: 468–478.
65. Walenski M, Mack JE, Mesulam MM, et al. Thematic Integration Impairments in Primary Progressive Aphasia: Evidence From Eye-Tracking. *Front Hum Neurosci*; 14. Epub ahead of print 6 January 2021. DOI: 10.3389/fnhum.2020.587594.
66. Wang H, Walenski M, Litcofsky K, et al. Online comprehension of verbal time reference in primary progressive aphasia: Evidence from eyetracking. *J Neurolinguistics* 2026; 78: 101318.
67. Watanabe Y, Takeuchi S, Uehara K, et al. Clinical availability of eye movement during reading. *Neurosci Res* 2023; 195: 52–61.
68. Wright IH, Sekar A, Jensen MT, et al. Reflexive and volitional saccadic eye movements and their changes in age and progressive supranuclear palsy. *J Neurol Sci*; 443. Epub ahead of print 15 December 2022. DOI: 10.1016/j.jns.2022.120482.
69. Zaino D, Serchi V, Giannini F, et al. Different saccadic profile in bulbar versus spinal-onset amyotrophic lateral sclerosis. *Brain* 2023; 146: 266–277.
70. Berry JD, Paganoni S, Carlson K, et al. Design and results of a smartphone-based digital phenotyping study to quantify ALS progression. *Ann Clin Transl Neurol* 2019; 6: 873–881.
71. Reilly J, Flurie M, Ungrady MB. Eyetracking during picture naming predicts future vocabulary dropout in progressive anomia. *Neuropsychol Rehabil* 2022; 32: 560–578.
72. Taylor JC, Heuer HW, Clark AL, et al. Feasibility and acceptability of remote smartphone cognitive testing in frontotemporal dementia research. *Alzheimer's and Dementia: Diagnosis, Assessment and Disease Monitoring*; 15. Epub ahead of print 1 April 2023. DOI: 10.1002/dad2.12423.
73. Armstrong MJ, Litvan I, Lang AE, et al. Criteria for the diagnosis of corticobasal degeneration. *Neurology* 2013; 80: 496–503.
74. Staffaroni AM, Clark AL, Taylor JC, et al. Reliability and Validity of Smartphone Cognitive Testing for Frontotemporal Lobar Degeneration. *JAMA Netw Open* 2024; E244266.
75. Anderson TJ, MacAskill MR. Eye movements in patients with neurodegenerative disorders. *Nature Reviews Neurology* 2013; 9: 74–85.

76. Filardi M, Gnoni V, Tamburrino L, et al. Sleep and circadian rhythm disruptions in behavioral variant frontotemporal dementia. *Alzheimer's and Dementia* 2024; 20: 1966–1977.
77. Merrilees J, Dowling GA, Hubbard E, et al. Characterization of apathy in persons with frontotemporal dementia and the impact on family caregivers. *Alzheimer Dis Assoc Disord* 2013; 27: 62–67.
78. Nagels G, Engelborghs S, Vloeberghs E, et al. Actigraphic measurement of agitated behaviour in dementia. *Int J Geriatr Psychiatry* 2006; 21: 388–393.
79. Paolillo EW, Casaletto KB, Clark AL, et al. Examining Associations Between Smartphone Use and Clinical Severity in Frontotemporal Dementia: Proof-of-Concept Study. *JMIR Aging*; 7. Epub ahead of print 2024. DOI: 10.2196/52831.
80. Tamburrino L, Tafuri B, Gnoni V, et al. Actigraphy validation in behavioral variant frontotemporal dementia. *Sleep Med* 2025; 126: 178–184.
